# Plant stress and proteomics in medicinal plants

**DOI:** 10.3389/fpls.2025.1656247

**Published:** 2025-09-30

**Authors:** Bita Kazemi Oskuei, Antonio Masi, Arkadiusz Kosmala, Nasser Mahna

**Affiliations:** ^1^ Department of Horticultural Sciences, Faculty of Agriculture, University of Tabriz, Tabriz, Iran; ^2^ Department of Agronomy, Food, Natural Resources, Animals and Environment, University of Padua, Agripolis, Legnaro, Padua, Italy; ^3^ Institute of Plant Genetics, Polish Academy of Sciences, Poznań, Poland

**Keywords:** biochemical pathways, medicinal plants, proteomics, stress, bioactive compounds

## Abstract

Medicinal plants serve as abundant reservoirs of natural compounds, including pigments, spices, insect repellents, and therapeutic compounds, which are utilized extensively in traditional systems. However, their phytochemicals, potential health benefits, and even response to extreme environments are not fully explored. A range of omics technologies has been extensively utilized in the study of medicinal plants to explore gene functions, unravel biosynthetic pathways of bioactive compounds, and understand the regulatory mechanisms behind gene expression. Due to the complex genetic regulatory network in medicinal plants, new technologies such as proteome assays make it easier to explain biological phenomena. Proteomics could offer a paradigm shift in our understanding of medicinal plants’ cellular metabolism. Until now, few classifications regarding recent and upcoming trends in proteomic studies in medicinal plants have been presented. This review highlights the most recent advances in medicinal plants’ proteomics and how proteomics gains insight into the dynamic changes in medicinal plants’ cellular metabolism.

## 1 Introduction

Since ancient times, diverse groups of people have used medicinal plants as primary remedies for the treatment and prophylaxis of numerous illnesses worldwide. Medicinal plants contain natural bioactive metabolites and compounds with strong therapeutic effects (Aye et al., 2019). Plant-derived antioxidants such as alkaloids, terpenes, polyphenols, and coumarins, secondary metabolites such as saponins and tanshinones, and specialized monomers like morphine, artemisinin, taxol, digitalin, and vinblastine constitute an essential part of primary substances for chemical drug development (Zhang et al., 2023).

At present, not only are many drugs obtained from different medicinal plants, but also a high number of the world’s population depend on traditional medicines for their primary healthcare. As a result, the global demand for herbal medicines continues to grow annually. In developed countries, comprehensive research is being conducted to isolate medicinal compounds from various species of medicinal plants and assess their biological activities (Zhang et al., 2023). However, the large-scale production of herbal medicines remains limited due to a lack of knowledge about the molecular basis of their metabolic processes.

The rapid development of modern high-throughput “Omics” approaches has introduced a series of breakthroughs in the identification of gene-protein-metabolite networks, novel biological metabolites with pharmaceutical properties, functional genes, molecular markers, as well as enzymes with a role in biosynthetic pathways in medicinal plants (Lifang et al., 2022). One of the most impressive Omics approaches applied to medicinal plants is proteomics, which recognizes a wide range of proteins involved in promoting and regulating biological processes. Proteomic analysis is considered an effective method for understanding regulatory mechanisms, providing cutting-edge information on the physiology and genetics of plants, and identifying proteinaceous compounds involved in the synthesis of bioactive compounds (Komatsu and Jorrin-Novo, 2021).

In recent years, the primary focus of proteomics in medicinal plants has been on identifying proteins with unknown or novel functions, their influence on metabolic pathways, their role in response to environmental stresses, and their function in the divergence of biological compounds (Zhang et al., 2023). Proteomic and phospho-proteomic analysis of differently cultivated *Dendrobium huoshanense* revealed changes in phosphorylation levels, as well as the localization of differentially accumulated proteins (DAPs) and phosphoproteins within the chloroplast. Moreover, the findings indicated that these proteins are involved in carbohydrate transport and metabolism, as well as the biosynthesis of secondary metabolites. In another study, the most affected pathways included signal transduction, linoleic acid metabolism, plant-pathogen interaction, phenylpropanoids biosynthesis, and the formation of transport barriers (Wu et al., 2022). Effective utilization of genetic diversity is the first step in developing medicinal plant varieties that can endure environmental stresses. The proteo-metabolomic reference map of *Paris polyphylla* varieties highlighted numerous components potentially linked to genotypic differences in medicinal constituents. These included upregulation of proteins associated with terpenoid backbone and steroid biosynthesis, effective sucrose utilization coupled with increased protein levels in the sugar metabolic pathway, and acetyl-CoA utilization efficiency in saponin biosynthesis, as identified using Sequential Window Acquisition of all Theoretical Mass Spectra (SWATH-MS) and GC/TOF-MS techniques. It was suggested that the interaction between genes, proteins, and metabolites plays a crucial role in the variability of medicinal compound content among genotypes (Liu F. et al., 2019).

According to Moyer et al. (2021a), medicinal plants used in traditional medicines are an excellent source of bioactive proteins and peptides that are not only antimicrobial but also part of the plant’s innate immune system, suggesting their dual role in both plant defense and potential human therapeutic applications. Cysteine-rich (Cys-rich) antimicrobial peptides (AMPs), as novel proteins/peptides that have pharmacological properties, found in trace amounts, and mostly underexplored, were discovered by bottom-up proteomic analysis of three edible traditional medicinal plants: *Trifolium pratense* (red clover), *Sesamum indicum* (sesame), and *Linum usitatissimum* (flax). These molecules with antimicrobial properties were classified into lipid transfer proteins, snakins, defensins, and α-hairpinins categories (Moyer et al., 2021b). The EF and MLH40 kind of anti-dengue virus (DENV) peptides that can attach to the E protein of DENV as the first line of the immune system, were identified from *Acacia catechu* medicinal plants using high-performance liquid chromatography (HPLC) (Panya et al., 2019).

Overall, research on medicinal plants has been greatly advanced by the rapid development and accessibility of analytical and computational approaches, particularly proteomics. Lao et al. (2014) conducted a comprehensive review of proteomic methodologies employed to elucidate therapeutic targets and molecular mechanisms underlying the pharmacological effects of Traditional Chinese Medicine (TCM). These approaches have facilitated the discovery of novel biomarkers, enabled the systematic characterization of numerous bioactive TCM compounds, supported the rational design of targeted therapeutics, and contributed to improved diagnostic precision across a spectrum of diseases (Zhao and Lin, 2014). Zaynab et al. (2018) examined the biochemical pathways responsible for synthesizing bioactive compounds in indigenous medicinal plants using proteomics. In another review, the recent developments of the biomarker investigation strategies and their imperative role in sustainable herbal drug developments were summarized. The altered proteins identified by proteomics can be introduced as potential drug targets to help comprehend a drug’s mechanism of action (Mumtaz et al., 2017). Aghaei and Komatsu (2013) also highlighted utilizing comparative proteomics to analyze the complex responses of medicinal plants to environmental stresses, which led to the discovery of their defensive mechanisms. These studies highlight the potential of an extensive proteomic dataset to enhance the effective utilization of medicinal plants. However, a thorough review of recent research progress in identifying proteins associated with medicinal plants remains absent. This review aims to address that gap by providing updated insights into the application of proteomic technologies in medicinal plant research, focusing on the discovery of bioactive compounds and their biosynthetic pathways for the production of natural drugs.

## 2 Proteomics studies in medicinal plants

### 2.1 Applied proteomic methodologies in medicinal plants

**Figure 1:**
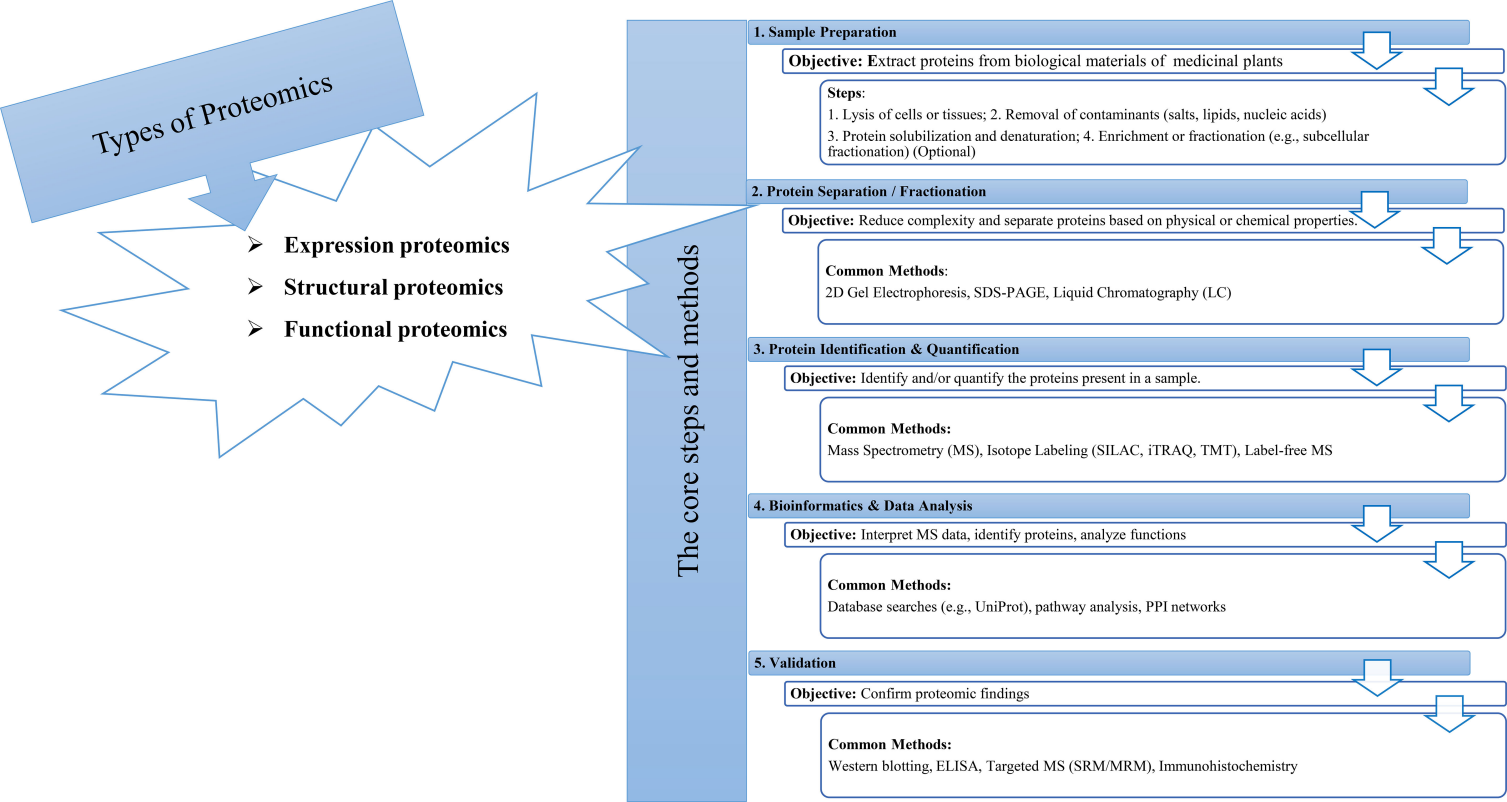
Proteomics: types, methods, steps.

**Figure 2:**
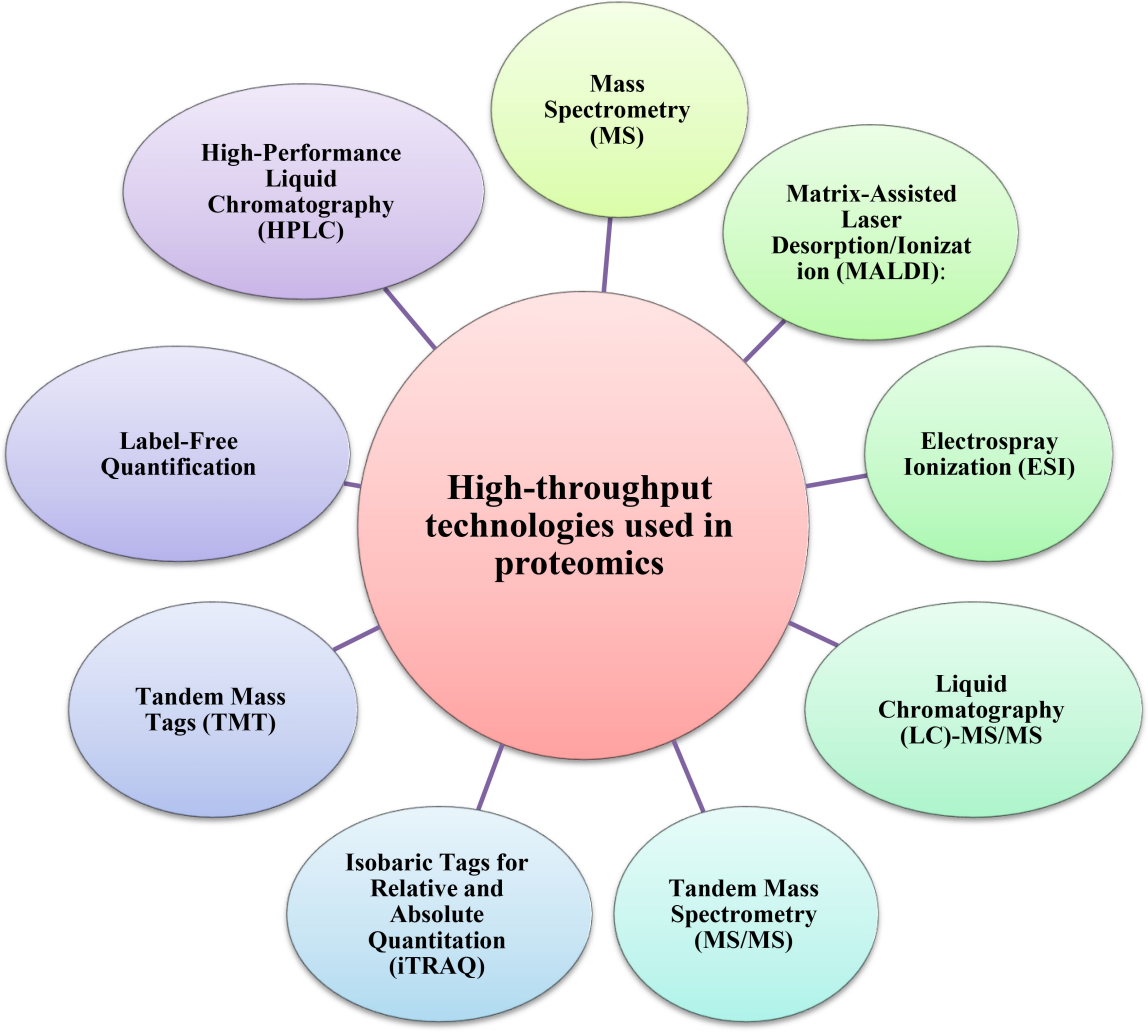
The most used high-throughput proteomics technologies for investigating medicinal plants.

The tools of proteomics are among the most important methods for understanding plant biological systems (**Figure 1**). Proteomic studies offer a large amount of information about the identity, quantitative profile, localization, and interactions of proteins in any of the specialized structures within a living cell of plants, including medicinal plants (Kumar and Kumari, 2018). Furthermore, the proteome analyses of medicinal plants enable researchers to identify systemic changes during cellular metabolism and associated pathways in bioactive compound production (Kim et al., 2016). Several high-throughput technologies have been developed to investigate the proteomes of medicinal plants in depth (**Figure 2**, **Table 1**).

**Table 1:** High-throughput proteomics technologies.

Technology	Abbreviation	Description	Advantages	Limitations
Mass Spectrometry	MS	Analytical technique that measures mass-to-charge ratio (m/z) of ionized particles	High sensitivity, accuracy, and dynamic range	Requires skilled operation, costly instruments
2D Gel Electrophoresis	2-DE	Separates proteins by isoelectric point and molecular weight	High-resolution separation	Limited to abundant proteins, labor-intensive
Matrix-Assisted Laser Desorption/Ionization	MALDI	A soft ionization method where a laser is used to ionize proteins embedded in a matrix	Tolerant to contaminants, good for large biomolecules	Limited to low-complexity mixtures, not ideal for quantification
Electrospray Ionization	ESI	Ionizes molecules by applying a high voltage to a liquid to create an aerosol	Gentle ionization, compatible with complex mixtures	Prone to signal suppression from salts and impurities
Liquid Chromatography–Tandem Mass Spectrometry	LC-MS/MS	Combines liquid chromatography with MS/MS to separate and analyze peptides	High resolution, can analyze complex samples	Expensive, time-consuming sample preparation
Tandem Mass Spectrometry	MS/MS	Sequential mass spectrometry; fragments selected ions to get structural information	Enables peptide sequencing and high specificity	Requires complex interpretation and processing
Isobaric Tags for Relative and Absolute Quantitation	iTRAQ	Chemical labeling technique for multiplexed quantification	Enables analysis of multiple samples in one run	Ratio compression, expensive reagents
Tandem Mass Tags	TMT	Similar to iTRAQ; allows simultaneous quantification of different samples	Highly sensitive, enables large-scale studies	Expensive, subject to interference
Label-Free Quantification	—	MS-based quantification without using chemical labels	Cost-effective, no need for labeling	Less precise than label-based methods
High-Performance Liquid Chromatography	HPLC	Separates proteins/peptides based on their physicochemical properties	High resolution, reproducibility	Not suitable for complex proteomes alone

Two-dimensional electrophoresis (2-DE) has been the leading technique for a long time to investigate the accumulation profiles of proteins in medicinal plants. However, its capabilities in protein identification and quantification were restricted. Although 2-DE could make comparisons easier, the accuracy of spot matching among a group of 2-DE gels was a tough task. Therefore, the 2-DE analysis was coupled with the mass spectrometry (MS) technology, which supplies higher resolution and improves reproducibility (Sharma, 2019). For example, Kim et al. (2016) employed a 2-DE gel analysis along with MS to identify proteins involved in redox regulation and contributing to the antioxidant activities in ginseng.

Later, second-generation proteomic techniques were developed to overcome the disadvantages of previous techniques. Isobaric tags for relative and absolute quantification (iTRAQ) is a sensitive non-gel-based quantitative proteomic technique that can be employed to evaluate differentially abundant proteins (DAPs). Liu et al. (2019) employed iTRAQ to reveal the causes of differences in secondary metabolites of Spica Prunellae (the fruiting spike of the perennial plant *Prunella vulgaris* L.), exposed to saline conditions. The iTRAQ uses isobaric reagents to monitor relative modifications in proteins and can provide more accurate quantification of DAPs, compared with the 2-DE gel approach. This approach has been extensively applied to study plant stress responses over specific time intervals. For example, Shi et al. (2017) successfully analyzed the protein profile of *Pyropia haitanensis* in response to different durations of high-temperature stress through iTRAQ. In addition, Li et al. (2017) used this method to reveal a picture of the damaging mechanisms due to replant disease stress in *Rehmannia glutinosa*.

Nowadays, high-throughput analysis of samples plays a key role in the investigation and modernization of traditional herbal medicine. Since quantification was not achievable by mass spectrometry, numerous strategies have been developed with distinct quantitation methods like labeling-based and label-free quantitation (**Table 2**).

**Table 2:** Different proteomic techniques, targets, and identified proteins in medicinal plants studies in the past two decades.

Analytical technique	Objective of the study	Targeted proteome		No. of identified proteins	Main identified proteins	Reference
		Medicinal species	Organ			
2-DE, ESI Q-TOF MS	High Light Response	*Panax ginseng* C. A. Meyer	Leaves	147	Cytosolic small heat-shock protein, cytosolic ascorbate peroxidase, putative major latexlike protein Rieske Fe/S protein, putative 3-beta hydroxysteroid dehydrogenase/isomerase-like protein, and oxygen-evolving enhancer-like protein	(Nam et al., 2003)
2-DE, MALDI-MS/MS	Alkaloid Biosynthesis	*Catharanthus roseus*	Cell suspension cultures	58	Two isoforms of strictosidine synthase, tryptophan synthase, 12-oxophytodienoate reductase	(Jacobs et al., 2005)
2-DE, MALDI-MS/MS	Ecotypic Variation	*Imperata cylindrica*	Leaves	4	Enolase, Mitochondria malate dehydrogenase, Ferredoxin-NADP(H) oxidoreductase	(Chang, 2008)
2-DE, LCMS/MS	Defense Responses	*Opium Poppy*	Cell cultures	219	SAM, trisophosphate isomerase, enolase, Chaperones, heat shock proteins, pathogenesis-related (PR) proteins	(Zulak et al., 2009)
2-DE, LCMS/MS	Changes in metabolism	*Eschscholzia californica*	Cell cultures	646	Methionine synthase, S-adenosyl methionine synthase, Glutathione-S-transferase, Phosphoenolpyruvate carboxylase	(Oldham et al., 2010)
Nano-LC-ESI-MS/MS	Medicinal properties	*Gynura procumbens (*Lour*.)* Merr	Leaves	92	Caleosin, Miraculin, Harpin protein, phosphate translocator	(Hew and Gam, 2010)
2-DE, MALDI-TOF/MS	Positive Allelopathic Stimulation Under Continuous Monoculture	*Achyranthes bidentata* Blume	Shoot	25	Chalcone synthase B, Acetyl-coenzyme A synthetase, RuBisCO activase, Auxin-responsive protein IAA9, Cysteinyl-tRNA synthetase Glutamine synthetase, Tubulin alpha-3 chain, Uridylate kinase, Recombination protein, Pyruvate dehydrogenase kinase	(Li et al., 2011)
2-DE, LC MALDI-MS/MS	(A)biotic stress response	*Nicotiana tabacum*	Trichomes	858	Superoxide dismutase, glutathioneS-transferase, peroxiredoxin, thioredoxin peroxidase, glutathione peroxidase, 1-hydroxy2-methyl-2-(e)-butenyl 4-diphosphate reductase	(Van Cutsem et al., 2011)
2-DE, MALDI TOF-TOF MS/MS	Methyl Jasmonate Elicitation Response Related To PTOX Accumulation	*Podophyllum hexandrum*	Cell suspension cultures	105	S-adenosyl-L-methionine-dependent methyltransferases, caffeic acid-O-methyl transferase, chalcone synthase, polyphenol oxidase, caffeoyl CoA 3-O-methyltransferase	(Bhattacharyya et al., 2012)
2-DE, MALDI TOF/TOF	Flavonoid and Phenylpropanoid Biosynthesis pathways	*Boesenbergia rotunda*	Cell suspension cultures	34	Caffeoyl-CoA-O-methyltransferase, fructose biphosphate aldolase, pyruvate kinase, pyruvate dehydrogenase, dihydrolipoyl dehydrogenase, glutamine synthetase	(Tan et al., 2012)
2-DE, MALDI-TOF MS	Flavonoid Accumulation Under Water Deficit	*Scutellaria baicalensis* Georgi	Root	24	Electron transporter, putative, Adenosylhomocysteinase, Chloroplast heat shock protein 70B, d-3-phosphoglycerate dehydrogenase, Alpha-tubulin	(Yuan et al., 2012)
2D-DIGE, MALDI-TOF/TOF	Comparative Proteomics	*Lycium barbarum* L	Anthers	45	Cysteine protease inhibitor, putative S-phase Kinase association Protein 1, aspartic protease, ascorbate peroxidase, putative glutamine synthetase, ATP synthase subunits, chalcone synthase, cysteine protease, carbohydrate metabolism-related, photosynthesis-related enzymes	(Zheng et al., 2012)
MALDI-MS/MS	Proteomic Analysis	*Mentha* sp*icata* L	Glandular trichomes	1666	Geranyl pyrophosphate synthase, geranyl geranyl pyrophosphate synthase, 4S-limonene synthase, (−)-(4S)-limonene-6-hydroxylase, pulegone reductase	(Champagne and Boutry, 2013)
2-D DIGE, nLC–MS/MS	Proteome Alterations Elicited With Methyl Jasmonate And Methyl B Cyclodextrin	*Silybum marianum*	Cell cultures	67	Pathogenesis-related proteins, proteins related to the transport process, proteins involved in metabolism, heat shock proteins	(Corchete and Bru, 2013)
2-DE, MALDI TOF-TOF MS	Comparative Proteomics	*Panax ginseng* CA May	Root	83	Cofactor-independent phosphoglyceromutase, Glyceraldehyde 3-phosphate dehydrogenase, Glutathione peroxidase, serine hydroxymethyltransferase 2, Heat shock protein Hsp70, Pterocarpan reductase Fructose-bisphosphate aldolas, 1,3-Beta-D-glucanase, Retrotransposon protein,	(Ma et al., 2013)
2-DE, MALDI TOF–TOF MSMS	Translational Plant Proteomics	*Mentha arvensis*	Leaves	59	HSP 70 kDa, DNA k-type molecular chaperone hsc70.1, Glutamine synthetase, Cytosolic ascorbate peroxidase, Carbonic anhydrase, Transcription factor NFE2 ATP synthase β chain, P0440D10.25, Oxygen evolving complex PS II 33 kDa pr (Sinha and Chattopadhyay, 2011),	(Sinha and Chattopadhyay, 2011)
2-DE, MALDI-TOF/TOF-MS	Proteomic Analysis Of Diploid Male Gametes Induced By Colchicine	*Ginkgo biloba* L	Microsporangia	27	Thiazole biosynthetic enzyme, glutamate-1-semialdehyde 2,1-aminomutase, heat shock protein 70 like protein, 26S proteasome ATPase subunit enolase2, Cytochrome c, Protein disulfide-isomerase, Elongation factor Tu 33 kDa oxygen-evolving protein, putative transketolase, NADPH thioredoxin reductase	(Yang et al., 2013)
2-DE, MALDI-TOF/TOF MS	Comparative Proteomic To Heat Stress	*Pinellia ternata*	Leaves	24	HSP21, heat shock protein 17.9, small heat shock protein 18.2 kDa class I hsp, glycine-rich RNA-binding protein GRP1A-like, glycine-rich RNA-binding protein	(Zhu et al., 2013)
SDS-PAGE, LC-MS/MS	Comparative Proteomic Of Mesophyll And Bundle Sheath Chloroplasts And Their Adaptation To Salt Stress	*Amaranthus cruentus*	Leaves	101	Deoxy-D-xylulose 5-phosphate reductoisomerase, chloroplastic, 5-Enol-pyruvylshikimate-phosphate synthase, Fructokinase, Salt tolerance protein I, Heat shock protein Hsp70 NADH-ubiquinone oxidoreductase 19 kDa subunit family protein, Chaperone, Heat shock protein,	(Joaquín-Ramos et al., 2014)
SDS-PAGE, nano-LC–MS/MS	Comparative Proteomic	*Chelidonium majus, Corydalis cava*	Shoot/tubers	16	Heat shock protein, Peroxidase, glutathione peroxidase, Cu/Zn superoxide dismutase, 14-3–3 protein, Lactoylglutathione lyase, putative/glyoxalase I,	(Nawrot et al., 2014)
SDS-PAGE, MALDI-TOF/TOF MS	Proteins Associated With Picroside Biosynthesis	*Picrorhiza kurroa*	Stolon	19	Glyceraldehyde-3-phosphate dehydrogenase, 1aminocyclopropane-1-carboxylate oxidase, photosystem I reaction center subunit V, 2-oxoglutarate ferrous-dependent oxygenase and putative cytochrome P450 superfamily protein	(Sud et al., 2012)
2-DE, MALDI-TOFMS	Proteins Induced By Salicylic Acid	*ginseng*	Suspension-cultured cells	15	Chaperonin cpn60, Methionine synthase, Malate dehydrogenase, Branched-chain-amino-acid aminotransferase-like protein 2-like, TUDOR-SN protein 1, Ribulose-1,5-bisphosphate carboxylase/oxygenase large subunit, Esterase D, putative, Predicted: elongation factor Tu, chloroplastic-like	(Sun et al., 2014)
2-DE, MS/MS	Establishing Proteome Reference Map	*Ginkgo biloba* L		158	Glutamine synthetase, Methionine synthase, Phosphoglycerate kinase, ATP synthase subunit alpha, Carbonic anhydrase, Ginnacin, Ascorbate peroxidase	(Uvackova et al., 2014)
iTRAQ-nano-HPLC-MS/MS	Mechanisms Of Embryo Abortion During Cross-Breeding	*Chrysanthemum morifolium*	Leaves	41	Cysteine synthase-like, Pathogenesis-related protein, Selenium-binding protein 2, Superoxide dismutase, Proteasome subunit alpha type, putative Tubulin alpha-6 chain, Beta-tubulin, UDP-glucose 6-dehydrogenase, Elongation factor EF-2, Putative S-adenosylmethionine synthetase	(Zhang et al., 2015)
2DE, LC-MS/MS	Elucidating The Biosynthetic Pathways Of The Artemisinin	*Artemisia annua*	Trichome	319	Ribulose-1,5-bisphosphate carboxylase/oxygenase large subunit, Cationic peroxidase 1-like, Chlorophyll a-b binding protein 40, chloroplastic-like, Peroxidase N1	(Bryant et al., 2015) Bryant et al.,2015
2DE, MALDI-TOF/MS	Proteomic Analysis	*Lagenaria siceraria*	Seed	24	Flavonoid 3′,5′-hydroxylase, Glyceraldehyde 3-phosphate dehydrogenase, RNA-directed DNA polymerase, Transferase, transferring glycosyl groups	(Kumari et al., 2015)
2-DE, MALDI-TOF/TOF	Adaptation Strategies At Altitude Gradient	*Potentilla saundersiana*	Leaves	118	(+)-Delta-cadinene synthase, Spermidine synthase 2, Soluble starch synthase 1, ATP synthase subunit a, Chalcone synthase D, Peroxidase 36, Indole-3-acetic acid-amido synthetase GH3.5, Heat shock 70 kDa protein 18 S-adenosylmethionine synthase, Glutamine synthetase cytosolic isozyme 1- 5, Beta-glucosidase 22, Oxalate oxidase 2, Strictosidine synthase	(Ma et al., 2015)
2-DE, MALDI-TOF-MS	Mechanisms Of Mycena Dendrobii Promoting Transplantation Survival And Growth Of Tissue Culture Seedlings	*Dendrobium officinale*	Root	41	Chalcone synthase, patatin-like protein 6, serine protease inhibitor, flavanone 3 β-hydroxylase, putative LOB domain protein 17, kelch repeat-containing F-box family protein, myrcene synthase-like protein	(Xu et al., 2015)
2-DE, MALDI-TOF MS	Postharvest Proteomic Changes Exposed To Enhanced UV-B Radiation	*Chrysanthemum morifolium* Ramat	Flowers	19	23 kDa thylakoid membrane protein, Glyceraldehyde 3-phosphate dehydrogenase, 60S acidic ribosomal protein P3, Ubiquitin-conjugating enzyme E2 35, Ascorbate peroxidase, Nucleoside diphosphate kinase, Alpha-barbatene synthase Ferrochelatase, Oxygen-evolving enhancer protein 1, chloroplastic, Formation of crista junctions protein 1, NADH-ubiquinone dehydrogenase, mitochondrial, Putative isoform 1, Elongation factor Tu, mitochondrial, GTP cyclohydrolase	(Yao et al., 2014)
2-DE, MALDI-TOF-TOF-MS	Proteomic Analysis Under Cadmium Toxicity	*Crocus sativus* L	Leaves	26	5methyltetrahydropteroyltriglutamate-homocysteine methyltransferase, Glutamine synthetase, S-adenosylmethionine synthetase 1, Glyceraldehyde-3-phosphate dehydrogenase Chaperonin, Stromal 70 kDa heat shock-related protein, chloroplastic, Ferredoxin-NADP reductase, leaves isozyme	(Rao et al., 2017)
2-DE, MALDI-QIT-TOF	Copper Excession Proteomic Change	*Hyoscyamus albus* L	Root	22	Pyrophosphate-fructose 6-phosphate 1phosphotransferasebeta-subunit, Enolase, S-adenosylmethionine synthase, Ferredoxin-nitrite reductase, Heat shock cognate 70 kDa protein Fructose-bisphosphate aldolase-like protein, Superoxide dismutase [Fe], Glutathione peroxidase, Proteasome subunit beta type-6	(Sako et al., 2016)
2D SDS-PAGE, MALDI-TOF/TOF-MS	Comparative Proteomic Of Male And Female Plants	*Simmondsia chinensis*	Leaves/male	45	Phosphoribulokinase, Plastidic aldolase/fructose-bisphosphate aldolase, Ribulose bisphosphate carboxylase, Glyceraldehyde 3-phosphate dehydrogenase, putative, Ribulose-1,5bisphosphate carboxylase/oxygenase large subunit Glyoxysomal malate dehydrogenase, Thioredoxin peroxidase, Ribulose-1,5bisphosphate carboxylase/oxygenase large subunit, Thioredoxin peroxidase, Chlorophyll a/b-binding protein	(Al-Obaidi et al., 2017)
2-DE, MALDI TOF/TOF MS/MS	Proteomic Change In Response To Alternaria Alternata Infection	*Withania somnifera* L	Leaves	38	ATP synthase subunit beta, ATP synthase epsilon chain, Acyl-[acyl-carrier-protein] desaturase, putatitve respiratory burst oxidase homolog protein, caffeoyl-CoA O-methyltransferase, apurinic endonuclease-redox, Sin3-like protein lipoyl synthase, adenylate isopentenyl transferase, cyclin-dependent kinase inhibitor, ferredoxinNADP reductase, Phenylalanine ammonia-lyase, putative disease resistance, apurinic endonuclease-redox	(Singh et al., 2017)
2-DE, MALDI-TOF/TOF-MS	Proteomic Analysis Of Purple Young Shoots During Leaves Development	*Camellia sinensis*	Leaves	46	Phosphoglycerate kinase, fructose-bisphosphate aldolase, sedoheptulose-1,7-bisphosphatase, malate dehydrogenase, pyruvate decarboxylase, glutamine synthetase, chaperonin family proteins Rubisco,	(Zhou et al., 2017)
2-DE, MALDI-TOF/TOF-MS	Comparative Proteomic Under UV Radiation	*Lonicera japonica* Thunb	Buds and Flowers	54	Sedoheptulose-1,7-bisphosphatase, 2,3-bisphosphoglycerate-independent phosphoglycerate mutase, transaldolase, alanine aminotransferase, 1-deoxy-D-xylulose 5-phosphate reductoisomerase, heat shock protein 70-2, Helicase, C-terminal, a ubiquitin-like protein oxygen-evolving enhancer protein 1-2, urease accessory protein G, 20S proteasome alpha subunit E2 isoform 2,	(Zhu et al., 2017)
SDS-PAGE, LC-MS/MS	Proteomic Analysis For Arsenic-Stress And Endosymbiosis	*Acacia farnesiana* L.	Shoot	81	Enolase (ch), PGK–like (ch), MDH, Succinyl–Coa ligase β–subunit (m), UDP–glucose pyrophosphorylase 1 (c), Adenosyl–homocysteinase [EC 3.3.1.1] (c), PPIase, chloroplastic, Mucunain (ch), Perchloric acid-soluble translation inhibitor–like (ch), GST [EC 2.5.1.18], amino-terminal domain (c) Predicted TPI (ch), pfkB family carbohydrate kinase (ext), Oxygen–evolving enhancer 2, chloroplastic–like, PPIase [EC 5.2.1.8] (c), Predicted PPIase–like (ch), APX, partial (c), Fe–SOD precursor [EC 1.15.1.1] (ch)	(Alcántara-Martínez et al., 2018)
2-DE, MALDI-TOF MS	Comparative Proteomic To An Altitudinal Gradient	*Herpetospermum pedunculosum*	Leaves	52	Lysosomal beta glucosidase-like isoform X1, enolase, ruBisCO large subunit-binding protein subunit beta, peptidyl-prolyl cis-trans isomerase CYP38, chloroplastic, copper-zinc superoxide dismutase ATP synthase subunit beta, mitochondrial, leucine aminopeptidase 1, protein PRUPE_ppa002167mg probable cytosolic oligopeptidase A, ribulose-1,5-bisphosphate carboxylase/oxygenase large subunit,	(Kim et al., 2018)
2-DE, MALDI TOF-TOF-MS	Comparative Proteomics In Response To Exogenous Glucose	*Lepidium draba* L		11	ATP-synthase CF1 alpha subunit, ribulose-1,5-bisphosphate carboxylase/oxygenase peroxisomal 2,4-dienoyl CoA reductase, Cytoplasmic aconitate hydratase, oxygen-evolving enhancer protein 1-2-chloroplastic-	(Rezaee et al., 2018)
2-DE, LC-MS	Comparative Proteomic Of Salt-Treated Natural Variants	*Imperata cylindrica* (L.) Beauv. var. major (Nees) Hubb	Leaves	20	Ribulose-1, 5-bisphosphate carboxylase/oxygenase small subunit, Photosystem I reaction center subunit IV, Hypothetical protein SORBIDRAFT_02g002690 (OEE2)	(Shih YunJhih et al., 2018)
2-DE, MALDI-TOF-TOF-MS	Impact Of Copper Stress On The Proteomic Behavior	*Eucalyptus camaldulensis*	Leaves	26	Elongation factor Tu, c-repeat binding factor, Glyceraldehyde-3-phosphate dehydrogenase, fructose-bisphosphate aldolase, sucrose synthase Ribulose bisphosphate carboxylase large chain,	(Alotaibi et al., 2019)
LC–MSMS	Somatic Embryogenesis (SE) Related Proteins	*Catharanthus roseus* (L.) G. Don	Hypocotyl	1079	40S ribosomal protein S12, 60S acidic ribosomal protein, ATP synthase subunit alpha, Catalase, Chaperone protein ClpB1, Elongation factor 1-alpha, Heat shock 70 kDa protein, Eukaryotic translation initiation factor 3 subunit D, Peroxidase	(Gulzar et al., 2021)
iTRAQ, Nano-LC-MS/MS	Proteomic Analysis Under Salt Stress	*Spica Prunellae*		35	60 S ribosomal protein L18a-2, 40 S ribosomal protein S3-2, 60 S ribosomal protein L6-2, pyruvate kinase, Glyceraldehyde-3-phosphate dehydrogenase, Cinnamyl alcohol dehydrogenase 2, Calcium-transporting ATPase, Calmodulin 5 60 S ribosomal protein, 60 S ribosomal protein L22-2, Nucleic acid-binding, OB-fold-like protein, Cytochrome c-2, Calmodulin 7, Nucleoside diphosphate kinase 1	(Liu Z. et al., 2019)
2−DE, MALDI-TOF/TOFMS	Proteome Analysis Under Water−Deficit Stress Induced By Polyethylene Glycol	*Lepidium draba*	Sprouts	20	Ribulose bisphosphate carboxylase/oxygenase activase A, partial, Oxygen-evolving enhancer protein 1–2, chloroplastic, Aminomethyltransferase, mitochondrial, Endopeptidase La Ribulose-1,5-bisphosphate carboxylase/oxygenase large subunit, partial (chloroplast), Peptidyl-prolyl cis-trans isomerase CYP38, chloroplastic	(Jamshidi Goharrizi et al., 2020)
LC-MS/MS	Proteome Analysis With Putative Anticancer Properties	*Viscum album* subsp	Leaves, stem, callus	5	Ribulose bisphosphate carboxylase large chain, Beta-galactoside-specific lectin 1 chain A isoform 1, Chitin-binding lectin, Beta-galactoside-specific lectin 3, Beta-galactoside-specific lectin 2	(Tsekouras and Kintzios, 2020)
LC-ESI MS/MS, iTRAQ	Proteomic Analysis Associated With Male Sterility	*Salvia miltiorrhiza*	Flowers	639	Malate dehydrogenase, pyruvate kinase, phosphoglucomutase, GAPDH, 26S proteasome,	(Wang et al., 2020)
2-DE, SDS-PAGE, MALDI-TOF/TOF	Proteomic Analysis Of Embryogenic Callus	*Panax ginseng* C. A. Meyer	Embryogenic callus	22	Glutamine synthetase, S-adenosyl-L-methionine synthetase 2, Malate dehydrogenase, Nucleotide-rhamnose synthase, ATP synthase b subunit, Heat shock protein, 14-3-3-like protein, Cinnamyl alcohol dehydrogenase S-adenosyl-L-methionine synthetase 1, Proteasome subunit beta type, Spermidine synthase	(Lei et al., 2021)
LC-MS/MS	High-Temperature Stress-Responsive Proteins	*Dendranthema grandiflorum*	Leaves	1463	Eukaryotic translation initiation factor 3, eukaryotic translation initiation factor 4B3-like, chloroplast translation initiation factor IF-2, fructose kinase	(Li et al., 2021b)
TMT labeling, LC-MS/MS	Meja Mediated Regulation Of Protostane Triterpene Biosynthesis.	*Alisma orientale* (Sam.) Juz.	Leaves	281	3-hydroxy-3-methylglutaryl-CoA reductase, squalene epoxidase, oxidosqualene cyclase, cytochrome P450s	(Rong et al., 2021)
iTRAQ, LC-MS/MS	Comparative Proteomic Analysis	*Paris polyphylla* var. *yunnanensis*	Seeds	1305	Adenylate kinase, Glutathione S-transferase, Isopentenyl-diphosphate Delta-isomerase I, Germacrene A synthase short form, E3 ubiquitin-protein ligase makorin, Probable cysteine proteinase At3g43960 precursor, NAD(P)H-quinone oxidoreductase subunit H, chloroplastic, Alcohol dehydrogenase 1, Receptor-like protein kinase HERK 1, 3-ketoacyl-CoA synthase 17, Cyclin-dependent kinase A-2	(Ling and Zhang, 2022)
SDS-PAGE, iTRAQ, MS	Proteomics Analysis Under Salt Stress	*Reaumuria soongorica*	Leaves	72	S-adenosylmethionine synthetase 1, Quinone-oxidoreductase homolog, Putative D-isomer specific 2-hydroxy-acid dehydrogenase, Chalcone–flavonone isomerase, CRS2-associated factor 2, chloroplastic, Ethylene-receptor, Auxin-responsive protein IAA11, Putative glycosyltransferase	(Yan et al., 2022)
LC-MS/MS	Proteomics for salinity resistance	*Portulaca oleracea*	Leaves	752	Glyoxylate and dicarboxylate metabolism, carbon fixation in photosynthetic organisms, cysteine and methionine metabolism, and glycolysis/gluconeogenesis	(Rodrigues Neto et al., 2023)
LC-MS/MS	Label-free proteomics salt stress	*Elaeagnus angustifolia*	Roots	4227	Aspartate aminotransferase, dehydratase-enolase-phosphatase 1 (DEP1), phospholipases D, diacylglycerol kinase, glycerol-3-phosphate O-acyltransferases, and gamma-glutamyl transpeptidases	(Chang et al., 2023)
UPLC-Q-TOF-MS analysis	Organ-specific proteomics	*Dipsacus asperoides*	Root, leaf, and flower	3,774	Acetyl Coenzyme a Acyltransferase, 3-Hydroxy-3-Methylglutaryl Coenzyme a Synthase, Hydroxymethylglutaryl-Coa Reductase, Mevalonate Kinase, Phosphomevalonate Kinase, Mevalonate Pyrophosphate Decarboxylase, Isopentenyl Diphosphate, Cytidylyltransferase, 4-(cytidine 50-diphospho)-2-C-methyl-D-erythritol kinase, 2-C-Methyl-D-Erythritol 2,4-Cyclodiphosphate Synthase; HDS, 4-Hydroxy-3-Methylbut-2-En-1-Yl Diphosphate Synthase, 1-Hydroxy-2-Methyl-2-(E)-Butenyl 4-Diphosphate Reductase, HMG-CoA, 3-Hydroxy-3-Methylglutaryl CoA,	(Pan et al., 2023)
2-DE, HPLC, LCMS/MS	Differential Regulation of Key Metabolism via Shotgun Proteomics	*neem (*Azadirachta indica*)*	Callus	129	Proteins associated with oxidoreduction, energy, transcriptional, stress response, respiration, and cell division	(Omar et al., 2024)

These promising approaches, including Liquid Chromatography with Tandem Mass Spectrometry (LC-MS-MS), and Matrix-assisted laser desorption/ionization time-of-flight mass spectrometry (MALDI-TOF MS), which offer high separation capacity and sensitivity, are the most commonly used analytical methods for the quantitative analysis of proteins in medicinal plants (Kang et al., 2022). Cheng et al. (2008) conducted a proteomic analysis using MALDI-TOF MS to investigate the anti-tumor mechanism of saponin in *Rhizoma Paridis* (*the rhizoma of Paris polyphylla* var. *yunnanensis (PPY) or P. polyphylla* var. *chinensis*). Proteomic analysis using LC-MS/MS revealed alterations in the proteome profile of medicinal plants traditionally employed as hypoglycemic agents for diabetes treatment, highlighting proteins associated with glucose regulation rather than insulin-like proteins (Pedrete et al., 2019). Proteins associated with energetic metabolic pathways and oxidative stress regulations have also been identified using LC-MS/MS in *Glycine max* (Kazemi Oskuei et al., 2017). Habib and Ismail (2021) demonstrated that about 60% of identified proteins by LC-MS/MS in *Phaleria macrocarpa* were essential components that regulate cell activity and enhance *P. macrocarpa*’s medicinal value.

Alternatively, the developments of MS-based proteomics have provided new opportunities for measuring proteins with critical contributions and elucidating mechanisms under different stress conditions (Liu et al., 2019b). Ma et al. (2022) identified a differential accumulation of proteins involved in the biosynthesis of flavonoids, alkaloids, phenylpropanoids, and amino acid metabolism via Tandem Mass Tag (TMT) based proteomic profiling of *S. alopecuroides* leaves under salt stress. The substantial accumulation of abiotic stress-associated proteins, as identified through MALDI-TOF MS, suggests that *Herpetospermum pedunculosum* employs a range of complex adaptive strategies in response to high-altitude environmental conditions. Furthermore, the observed association between these stress-related proteins and those involved in photosynthesis indicates a potential functional interplay contributing to the plant’s acclimation mechanisms (Kim et al., 2018).

Proteomics relies on the availability of genomic sequences essential for protein identification using bioinformatic tools. Matching predicted sequences with analytically obtained spectra is a fundamental step in proteomics, made possible by genomic sequencing projects conducted worldwide in recent years. By the end of 2020, more than 1000 genomes of different plant species had been sequenced, but this number has been growing exponentially (Sun et al., 2022). Most of these species have agronomic and economic importance, but only a few are medicinal plants. Genome sequencing of many medicinal plants is underway (Pei et al., 2024), but the number of plants with potential medicinal uses is extremely large. This constitutes a substantial constraint in the execution of proteomic analyses; nevertheless, RNA sequencing technology presents a promising and cost-efficient alternative for mitigating this limitation. Therefore, the MS data can be searched against the newly obtained RNAseq database, thus expanding the possibility of performing a proteomic analysis on virtually any plant species.

### 2.2 Proteomics and metabolic pathways

Highly conserved metabolic pathways are extensive networks of biochemical reactions necessary to maintain and regulate life activities. Comprehending metabolic pathways requires the systematic study and accurate mapping of the biochemical processes without considering dead-end reactions or futile loops. Metabolic pathways analysis provides critical insights into the flow of metabolites, the different regulation of reactions, and the various pathways (Altaf, 2016). Plants exploit metabolic systems for many kinds of bioactive compounds. Although multiple metabolites may have very similar chemical structures/polarities, they may utilize different substrates to construct different products. Hence, quantitative information on every metabolite from multiple pathways is needed. Proteomics and metabolomics approaches provide solutions to discern important metabolic pathways and metabolites’ assays in a wide range of biological samples. In other words, proteomic techniques enable the identification of proteins, including enzymes, the synthesis and functions involved in plants’ primary/secondary metabolism, the measurement of systemic changes during cellular metabolism, and the evaluation of bioactive compounds biosynthesis, which confer pharmacological effects on medicinal plants.

Proteomics analysis of *Pyropia haitanensis* has been effectively utilized to identify proteins involved in the phosphoinositide pathway, which is involved in signal transduction. This includes the up-regulation of specific proteins associated with glycolysis, the citric acid cycle, and beta-oxidation of fatty acids (Shi et al., 2017). TMT-based proteomic analysis has been used by Vidović et al. (2023) to describe the metabolic pathways involved in *Ramonda serbica Panc*. desiccation tolerance, so that proteins and transcripts linked to late embryogenesis abundant proteins, C1 metabolism, folding and assembly, protein production, heme synthesis, nitrogen metabolism, and fermentation showed higher levels of accumulation. The pathways that produce essential metabolites must be composed of enzymes and proteins with high variation in abundance that control metabolic fluxes, substrate utilization, and product agglomeration. Thus, considering these concepts can lead to predicting the activity and bottlenecks of key metabolic pathways and facilitate the development of strategies to ameliorate crops by enhancing secondary metabolite production. For instance, 4-hydroxyisoleucine (4-HIL), as the main biologically active compound in *Trigonella foenum-graecum* L. (fenugreek), was increased through metabolic engineering (Safari et al., 2020). Niu et al. (2021) indicated that both mevalonate kinase and phosphomevalonate kinase were the potential bottleneck proteins in the regulatory mechanisms and were involved in the mevalonic acid pathway, which might contribute to the biosynthesis of sesquiterpenes in *Santalum album.* In the model grass *Brachypodium distachyon*, ammonia-lyases (ALs) and lignin biosynthetic protein families were found to be the most abundant proteins in lignified tissues. Additionally, changes in metabolomic and proteomic data highlighted crosstalk between lignin biosynthesis and primary metabolisms, particularly nitrogen metabolism (Barros et al., 2022). 2-DE coupled with MS revealed significant changes in the intensity of proteins in response to homocysteine and suggested that the secondary metabolites of *Salvia miltiorrhiza* inhibited homocysteine-induced A10 cell growth via the PKC/MAPK-dependent pathway (Hung et al., 2010). Understanding the biosynthetic pathways of ginsenosides, a class of triterpene saponins that are almost exclusively found in ginseng, is a challenge owing to ginseng’s long life cycle. Investigating metabolic fluctuations along with ginseng growth through proteomics revealed the positive correlation of proteins with a ginsenoside in roots of ginseng, and ginsenoside biosynthesis pathways commence when the ginseng (*Panax ginseng*) reaches a slow-growth period (Li et al., 2021a). In *Artemisia annua*, a series of enzymatic pathways, including peroxidases within the trichome-specific proteome, were shown to play an effective oxidative reaction role in the final stages of artemisinin biosynthesis, which have so far been thought to be non-enzymatic in nature (Bryant et al., 2015). According to Decker et al. (2000), codeinone reductase, the most abundant detected protein by proteomic analysis using 2-DE, was guessed to be a specific enzyme involved in morphine biosynthesis in poppy latex (*Papaver somniferum*).

Significant up-regulation of ABA-responsive protein, ATP-dependent fructose 6-phosphate kinase, late embryonic development abundant protein_2 (LEA_2) domain-containing protein, α-galactosidase, and (Heat-Shock Protein) HSP family proteins after severe drought stress was observed in highly drought-tolerant Licorice (*Glycyrrhiza uralensis*) using quantitative proteomics with TMT tagging combined with liquid chromatography-tandem mass spectrometry (LC-MS/MS). Some of these DAPs that induced stress tolerance in licorice were detected to be enriched in a large number of secondary metabolism-related pathways after drought exposure, including flavonoid, terpene skeleton, sesquiterpene, carotenoid, and phenol propane biosynthesis, as well as amino acid, sugar, and lipid metabolisms (Zhang et al., 2022). *Prunus mira* medicinal plant, after osmotic stress, induced an adaptive mechanism by influencing proteins related to energy metabolism, photosynthesis, carbohydrate metabolism, transport, translation, molecular chaperones, stress, and defense. Thus, these proteins’ abundance enhancement and lower energy accumulation contributed to maintaining a balance of metabolites and dealing with abiotic stress (Xu et al., 2021). The regulation of the mechanisms underlying haustorium development in the *Taxillus chinensis* medicinal plant is imperative for its successful parasitic invasion. Based on quantitatively iTRAQ-based proteomics analysis, upregulation of crucial proteins involved in the phenylpropanoid metabolic pathway and proteins associated with ABA signaling that act as inhibitors for regulating ABA biosynthesis, induce lignin accumulation and keep the levels of ABA down for improving haustorial development (Pan et al., 2021). Nawrot et al. (2017) suggested that during plant development, biological activity transitions from vigorous biosynthetic processes to the activation of defense mechanisms.

### 2.3 Proteomics for finding bioactive compounds in medicinal plants

Most medicinal plants contain beneficial bioactive compounds that are considered potential therapeutic agents and precursors for drug biosynthesis. However, their pharmacological activities and biosynthetic pathways remain unidentified (Hashiguchi et al., 2017). To effectively utilize plants’ genetic resources and metabolic products, conducting physiological studies is vital, especially concerning secondary metabolism and the pharmacological effects of plant-derived compounds on humans and animals. MS techniques provide detailed insights into secondary metabolites, their intricate biosynthetic pathways, and the external factors influencing these processes. Johnson et al. (2001) employed this strategy to screen toxic rosmarinic acid and electrophilic quinoid metabolites from plant extracts. Pulsed ultrafiltration and LC-MS/MS analyses demonstrated that *Trifolium pratense*, known for its metabolites used in treating menopausal symptoms, does not produce glutathione (GSH) adducts of toxic reactive metabolites. In contrast, *Symphytum officinale*, *Sassafras albidum*, and *Rosmarinus officinalis*, recognized for their carcinogenic or toxic compounds, were found to generate GSH adducts along with a newly identified quinone metabolite, rosmarinic acid.

Comparative proteomics of *Cannabis sativa* as a herbal medicine was done to determine tissue-specific accumulation of proteins and enzymes involved in cannabinoid biosynthesis. Most of the identified protein spots were found to be associated with primary metabolism, whereas only a single protein related to cannabinoid biosynthesis was detected. This may be attributed to the low abundance of proteins involved in secondary metabolism, such as those involved in cannabinoid biosynthesis (Raharjo et al., 2004). Post-translational modifications played a significant role in the proteomes of *Cannabis* flowers, with various modified proteins showing potential connections to the production of terpenes and cannabinoids, the primary bioactive compounds in *Cannabis* (Jenkins and Orsburn, 2020). DAPs from *Eruca sativa* and *Linum usitatissimum* that were identified by top-down proteomics had a good range of biological functions, including antimicrobial, anti-aphid, antigenic, and cardio-protective effects. These bioactive peptides belong to different classes of AMPs and have the potential against life-threatening diseases, so they could be used in drug development (Altaf, 2016).

The alterations in the *Panax ginseng* root protein and transcript profiles correlated with the activity of enzymes within up- and downstream processes of the ginsenoside biosynthesis network in different stages (Jayakodi et al., 2015). Ginsenosides are a class of triterpene saponins that are exclusively found in *Panax spp* as bioactive components. *Panax ginseng* herb is used to treat physiological disorders. Studies have shown that ginseng promotes health and prevents diseases (Rajabian et al., 2019), including immune modulation, anti-inflammatory effects, lipid-lowering, antioxidation, anti-diabetic, anti-tumor activities, increased energy, restorative properties, anti-aging, anti-depression, inhibition or delay of the neurodegenerative process, and improvement of memory and perceptual systems (Ong et al., 2015). Using 2-DE and mass spectrometry (GC-TOF MS) led to the identification of DAPs related to the hydrolase, oxidoreductase, and ATP-binding activities. Subsequently, extensive identification of metabolites with potential beneficial health effects, including amino acids, sugars, organic acids, phenolic acids, phytosterols, tocopherols, and policosanols, pointed to the occurrence of extremely active biosynthetic pathways of medicinal compounds in *Panax ginseng* (Kim et al., 2016). DAPs identified by proteomics analysis indicated the interaction of metabolic proteins associated with the growth strategies. The major part of identified proteins was affiliated with energy metabolism and were used to store energy for promoting root elongation and thickening, stress resistance, as well as improving the biosynthesis of the secondary metabolites, such as ginsenoside biosynthesis (Ma et al., 2013).

A diverse array of secondary metabolites, produced via specific metabolic pathways, is frequently activated by environmental stimuli. Zheng et al. (2016) reported that short-term, high-dose UV-A radiation activated the stress defense system in *Taxus chinensis* and enhanced the production of the anticancer metabolite paclitaxel. Gel-based proteomics and GC-MS analyses of its leaves and chloroplasts revealed that UV-A radiation predominantly impacted systems related to photosynthesis, glycolysis, secondary metabolism, stress response, protein synthesis, degradation, and activation. Additionally, the upregulation of four key glycolysis enzymes, along with 1-deoxy-D-xylulose-5-phosphate reductoisomerase and 4-hydroxy-3-methylbut-2-enyl diphosphate reductase, provided essential precursors for secondary metabolism, leading to increased paclitaxel production through activation of its biosynthetic pathway. Proteome analysis (Q-TOF–LC-MS/MS) of the jasmonic acid-treated *Andrographis paniculata* (green chiretta) medicinal plant revealed induction of protein accumulation involved in the isoprenoid pathway, terpenoid biosynthesis, and andrographolide production. Functional annotation and KEGG analysis of these DAPs unveiled highly elevated metabolic processes as well as secondary metabolism-related proteins associated with phenylpropanoids, isoprenoids, and flavonoid pathways. The enhancement of andrographolide production was found to contribute to the upregulation of terpenoid biosynthesis (Bindu et al., 2020).

### 2.4 Proteomics of response to biotic stresses in medicinal plants

Pests, parasites, and pathogens are responsible for a large number of plant infections. Feeding on a living host or killing it through toxin secretion, vascular wilt induction, leaf spots, stunted growth, and wilting are part of their capacities to affect plants and induce biotic stresses. Viruses led to systemic damage, resulting in stunting and chlorosis, while mites and insects impair plants by laying eggs on them. Combating such stresses demands an elaborate immune system in plants. Cuticles, wax, and trichomes are physical barriers that plants use as the first defense line. At the cellular level, producing chemical compounds is one of the plants’ capabilities to defend themselves. In response to pathogen attacks, plants activate mitogen-activated protein kinases (MAPKs), volatile compounds, PAMP-triggered immunity (PTI), generating reactive oxygen species (ROS) and Effector-triggered immunity (ETI). Induced ETI and PTI influence specific downstream signaling pathways, including salicylic acid (SA) regulatory pathways. Additionally, hydrogen peroxide (H_2_O_2_), activated oligogalacturonoids (OGAs), and jasmonic acid (JA) signaling pathways are components of plant systemic defense responses (Iqbal et al., 2021).

Another pivotal downstream defense mechanism of plants is the generation of defensive and stress proteins comprising α-amylase inhibitors, protein inhibitors, polyphenol oxidases, chitinases, lectins, and PR proteins (Chi et al., 2019; Perlikowski et al., 2019).

There is substantial evidence that variations in the proteome and post-translational modifications (PTMs) play a direct role in the plant immune response. Thus, for a comprehensive understanding of medicinal plant responses to biotic stresses, proteomics assessment is crucial. Soybean extracts are popularly known as medicinal ingredients. Dong et al. (2015) conducted a proteomic analysis using 2-DE to investigate soybean innate immunity in response to *Bipolaris maydis* inoculation. Their findings demonstrated a systematic Nonhost Resistance (NHR) against non-adapted pathogens, activating major metabolic processes, subcellular structures, and multi-gene resistance mechanisms. Key proteins such as acidic chitinases, protease, Kunitz-type protease inhibitors (PI), RuBisCO (Ribulose-1,5-bisphosphate carboxylase/oxygenase), BAHD (Benzyl alcohol acetyltransferase family), NDK (Nucleoside diphosphate kinase), and OEE (Oxygen-evolving enhancer protein) played significant roles in soybean NHR, with some overlapping metabolic functions.

Additionally, proteomic changes assessed via 2D-PAGE and LC-MS/MS during the biotrophic phase of *Theobroma cacao* L. genotypes revealed differential responses to *Moniliophthora perniciosa* infection. Several altered proteins were linked to essential biological functions in resistance, including oxidative stress regulation, photosynthesis, carbohydrate metabolism, and detoxification. The upregulation of chitinases, trypsin inhibitors, and PR 5 proteins as defense and stress-related proteins was enhanced in both resistant and susceptible inoculated genotypes, reinforcing their roles in fungal resistance (dos Santos et al., 2020).

Proteomic analysis of strawberry seedling cell responses to *Colletotrichum fragariae* infection, utilizing 2-DE and MALDI-TOF/TOF MS/MS, identified proteins with significant abundance variations. The infection led to an increase in β-1,3-glucanase, low-molecular-weight heat shock proteins, and novel pathogen-responsive proteins, whose expression patterns correlated with physiological changes. Conversely, the infection suppresses proteins involved in the Calvin cycle and glycolysis pathway, affecting metabolic processes. In consequence, a pathogen-responsive protein network was induced (Fang et al., 2012).

In several pathosystems, infected plants or hosts reduce their photosynthetic rates to mobilize energy for defense responses and carbohydrate assimilation. The up-regulation of proteins related to carbohydrate metabolism refers to the required respiration enhancement. Induced photorespiratory pathway represents a high source of photosynthesis-related-ROS during infections; this has an important role in retrograding signaling pathways and leading to biotic stress defense-related genes’ expression, which increases, eventually, the hypersensitive response (HR) (Souza et al., 2019). During compatible and incompatible interaction of Mungbean Yellow Mosaic India Virus (MYMIV) infection with *Vigna mungo*, which can cure several diseases, biochemical and comparative proteomic analyses deciphered differential regulations of V. mungo leaf proteome upon MYMIV infection and elucidated its resistance response mode at the biochemical level. It was indicated that photosynthesis-related proteins and proteins involved in energy metabolism were mostly affected, resulting in reduced photosynthesis rate and correlating with the appearance of disease symptoms. Key factors in evoking the MYMIV-resistance mechanism were, namely the accumulation of proteins related to signal transduction, ROS metabolism, defense/stress, and redirecting carbohydrate flux toward the pentose phosphate pathway (Kundu et al., 2013).

Peptide-level evidence identified seven novel antimicrobial peptides (AMPs) distributed across three distinct AMP classes: snakins, defensins, and lipid transfer proteins, and unclassified putative AMPs were characterized by a Bottom-up LC-MS/MS-based proteomics/peptidomics analysis in edible amaranth (*Amaranthus tricolor*) plants. Isolated Atr-SN1, Atr-DEF1, and Atr-LTP1 as short-chain proline-rich antibacterial peptides demonstrated activity against the high-risk ESKAPE bacterial pathogens and further suggested that many unknown bioactive peptides with potent inhibition activities remain to be discovered (Moyer et al., 2021a).

### 2.5 Proteomics of response to abiotic stresses in medicinal plants

#### 2.5.1 Salinity

Salinity poses a significant challenge to sustainable agriculture by disrupting plant growth and development. It primarily exerts its negative effects by extracting water from the cytoplasm, leading to osmotic stress (Akula and Ravishankar, 2011). Osmotic stress disrupts metabolic balance and leads to the accumulation of harmful reactive oxygen species (ROS), exacerbating cellular damage and impairing plant function (Younessi-Hamzekhanlu et al., 2021; Zhao et al., 2021), induces specific secondary metabolites production, stimulates the activity of ROS scavenging enzymes (Huang et al., 2020), and affects photosynthesis through destabilizing the chloroplast structure, chlorophyll biosynthesis, and eventually photosynthetic rate (Hnilickova et al., 2021). Plants respond to salinity by modulating various morphological, physiological, and biochemical traits by regulating ion homeostasis and biosynthesis of osmoprotectants, antioxidants, and phytohormones (Arif et al., 2020). Utilization of the impressive proteomics technologies could provide noteworthy information about the complex impact of stress on medicinal plants along with stress-tolerance mechanisms concerning photosynthesis, ion homeostasis, ROS signaling, protein phosphorylation, osmotic modulation, signaling transduction, and post-translational regulation (Zhang et al., 2023).

In general terms, it is believed that the process of photosynthesis is sensitive to salinity. Up-regulation of Rubisco activase, Rubisco large subunit, and carbonic anhydrase as photosynthetic proteins was observed in the proteomic response of medicinal halophyte *Limonium bicolor* (bicolored sea lavender) leaves under salt stress. The elevated abundance of these proteins, which boosted photosynthesis levels, played a crucial role in enabling *Limonium bicolor* to withstand salt stress (Wang et al., 2017). In *Aeluropus lagopoides*, salt stress led to the downregulation of Rubisco’s small subunit at the protein level (Sobhanian et al., 2010). Similar results have also been reported in association with photosynthetic proteins including Rubisco small subunit, LOC100194054, Cytb6-f, oxygen-evolving enhancer with differential accumulation patterns in response to salt stress in *Imperata cylindrica* (L.) medicinal plant (Shih YunJhih et al., 2018). With a focus on the effect of salt stress on proteins of chloroplasts in *Amaranthus cruentus* as a photosynthetic C3-C4 medicinal plant by LC-MS/MS, Joaquín-Ramos et al. (2014) also reported that salt stress triggered alterations in the proteomic profiles of thylakoid protein complexes in both bundle sheath cells and mesophyll cells were induced by salt stress; in addition, enhancement of ATP-synthase and the CYTb6f proteins were associated with a significant demand for ATP during the salt stress response.

High ATP utilization in the leaves of *Morus alba* L. under salt stress resulted in an increased NADPH/ATP ratio, thereby enhancing cyclic electron flow (CEF) (Huihui et al., 2020). Reduced ATP synthesis plays a role in minimizing photoinhibition (Takagi et al., 2017). TMT-based proteomics analysis revealed that mulberry seedlings exposed to NaCl and NaHCO_3_ stress exhibited disruptions in internal photoprotective mechanisms. The down-regulated xanthophyll cycle, cyclic electron flow (CEF), chlorophyll synthesis, Fd-dependent ROS metabolism and nitrogen metabolism due to decrease in abundance of the LHCII antenna (CP24 10A, CP26, and CP29), PSII oxygen-evolving complex (OEE3–1 and PPD4), and photosystem I core proteins (PsaF, PsaG, PsaH, PsaN, Ycf4) inhibited photosystem electron transfer and carbon assimilation. Therefore, the main prerequisite to keeping plants’ photosynthetic function and improving plant tolerance under stress conditions was photoinhibition as an effective regulatory mechanism (Huihui et al., 2020).

Plants activate diverse mechanisms to counteract salinity, including the stimulation of protective and antioxidant enzymes. A systematic proteomic analysis of *Andrographis paniculata* under salinity stress, employing a combination of 2-DE and MS, identified proteins that were upregulated and downregulated in response to stress. These proteins, which contributed to stress protection, were associated with superoxide dismutase, ascorbate peroxidase, and ribulose-1,5-bisphosphate oxygenase, functioning as ROS scavengers (Talei et al., 2015). It was suggested that *Limonium bicolor* leaves high stress-tolerance was due to its great constitutive antioxidant and detoxification capacity (Wang et al., 2017).

The root proteome profiling of *Pongamia pinnata* under salt stress was performed by employing the free-labeled nanoLC-MS/MS method, showing an elevated presence of proteins associated with essential regulatory pathways, including chalcone synthase (CHS) proteins, which take part in flavonoid biosynthesis, were involved in signaling pathways, including secondary metabolism, anaerobic respiration, and antioxidant metabolism associated with salt tolerance. Moreover, the significant abundance of CHS proteins has contributed to adjusting ROS cellular energy redox homeostasis against Na^+^ toxicity with high antioxidant activity under saline environment conditions (Marriboina et al., 2022).

Plants activate a series of responses and defense mechanisms facing abiotic stresses to transmit signals and increase tolerance (**Table 3**). The comparative proteomic analyses of *Limonium bicolor* leaf and root exposed to salt stress utilizing 2D-PAGE combined with MALDI-TOF/TOF-MS revealed DAPs with diverse physiological functions spanning a broad spectrum of molecular processes leading to salt-stress adaptation and some chemical constituent enhancement by activating multiple biological pathways including energy, secondary metabolism, redox homeostasis, carbohydrate, transcription, and transport (Wang et al., 2017). iTRAQ-labeled quantitative proteomics in *Helianthus tuberosus* L. under salt stress revealed that DAPs were predominantly enhanced in redox regulation, carbohydrate metabolism, ion binding processes, and ribosome translation (Zhang et al., 2018). A rise in soluble carbohydrates, restructuring of ribosomes, and elevated levels of enzymes participating in the citrate cycle, glycolysis, and the pentose phosphate pathway, with an impact on plant developmental processes and controlling organelle trafficking energy resource could conduct modulation of the plant metabolism in salt defense (Li et al., 2015). Hence, the induction of sugar signaling proteins and ribosome activity was introduced as the reason for the salt tolerance appearance in *H. tuberosus* (Zhang et al., 2018). The utilization of (iTRAQ) MS/MS by Liu Z. et al. (2019) significantly enabled the detection of the changes in proteins and related molecular mechanisms in *Spica prunellae*, a Chinese traditional herb under different salt concentrations. Salt-stressed *Spica prunellae* activated a bunch of mechanisms by enhancing proteins related to energy metabolism, protein metabolism, photosynthesis, and oxidative capacity that afford tolerance to salt stress.

**Table 3:** Different proteomic techniques, targets, and identified proteins of various medicinal plants under abiotic stresses.

Categories of stress	Type	Species of the study	Organ		Analytical technique	Main identified proteins	Reference
Salinity	Salt Stress	*Amaranthus cruentus*		Leaves	SDS-PAGE, LC–MS/MS	Thylakoid proteins	(Joaquín-Ramos et al., 2014)
	Salt Stress	*Andrographis* *paniculata* Nees		Leaf/ root	2-DE, MALDI TOF MS	Glutamine synthetase, Methionine synthase, Phosphoglycerate kinase, ATP synthase subunit alpha, Carbonic anhydrase, Ginnacin, Ascorbate peroxidase	(Talei et al., 2015)
	Salt Stress	*Helianthus tuberosus* L.		Leaf	iTRAQ-labeled quantitative proteomics	Redox regulation, carbohydrate metabolism, ion binding processes, and ribosome translation	(Zhang et al., 2018)
	Salt Stress	*Spica Prunellae*		Leaf	iTRAQ, Nano-LC-MS/MS	60 S ribosomal protein L18a-2, 40 S ribosomal protein S3-2, 60 S ribosomal protein L6-2, pyruvate kinase, Glyceraldehyde-3-phosphate dehydrogenase, Cinnamyl alcohol dehydrogenase 2, Calcium-transporting ATPase, Calmodulin 5, 60 S ribosomal protein, 60 S ribosomal protein L22-2, Nucleic acid-binding, OB-fold-like protein, Cytochrome c-2, Calmodulin 7, Nucleoside diphosphate kinase 1	(Liu et al., 2019)
	Salt Stress	*Morus alba L.* *(mulbe*rry)		Seedlings	TMT-based proteomics	Down-regulated xanthophyll cycle, cyclic electron flow (CEF), chlorophyll synthesis, Fd-dependent ROS metabolism and nitrogen metabolism, LHCII antenna (CP24 10A, CP26, and CP29), PSII oxygen-evolving complex (OEE3–1 and PPD4), and photosystem I core proteins (PsaF, PsaG, PsaH, PsaN, Ycf4)	(Huihui et al., 2020)
	Salt Stress	*Medicago sativa* *Medicago truncatula*		Root	2-DE, MALDITOF/TOF-MS	*APR* and *COMT*	(Long et al., 2016)
	Salt Stress	*Pongamia pinnata*		Root	Free-labeled nanoLC-MS/MS	Chalcone synthase (CHS) proteins, flavonoids biosynthesis, signaling pathways, secondary metabolism, anaerobic respiration, and antioxidant metabolism	(Marriboina et al., 2022)
	Salt Stress	*Reaumuria soongorica*		Leaf	SDS-PAGE, iTRAQ, MS	Photosynthesis (RCA, PSAF, PSAN, PSB27-1), ribosomal proteins (RPS10, RPS2D, RPS9, RPL7AA), peptide chain-releasing factor (AT2G47020)	(Yan et al., 2022)
Light spectrum	Dark Stress	*Picrorhiza kurrooa* Royle ex Benth.		Leaf/root	2-DE, MALDI-ToF/ToF-MS	MLP-like protein 31 (MLP31) 1,2-Dihydroxy-3-keto-5methylthiopentene dioxygenase, OTU domain-containing protein At3g57810, Cytokininriboside 50monophosphate phosphoribohydrolase LOG2 Cold shock domain-containing protein 4, DNA-directed RNA polymerase subunit alpha, Protein vernalization insensitive 3 Protein transport protein Sec 61 subunit gamma-3, Plastid lipidassociated protein 1 50S ribosomal protein	(Parkash et al., 2014)
	Uv-B Radiation	*Catharanthus roseus*		Leaf	LC-MS/MS	Sedoheptulose-1, 7-bisphosphatase, chlorophyll a/b binding protein, serine glyoxylate aminotransferase 3, ribulose bisphosphate carboxylase/oxygenase, and light-harvesting complex II proteins comparative proteomic	(Zhu et al., 2015)
	Uv-B Radiation	*Chrysanthemum morifolium* Ramat		Flowers	2-DE, MALDI-TOF MS	23 kDa thylakoid membrane protein, Glyceraldehyde 3-phosphate dehydrogenase, 60S acidic ribosomal protein P3, Ubiquitin-conjugating enzyme E2 35, Ascorbate peroxidase, Nucleoside diphosphate kinase, Alpha-barbatene synthase Ferrochelatase, Oxygen-evolving enhancer protein 1, chloroplastic,	(Yao et al., 2014)
	Uv-B Radiation	Ginkgo biloba		Leaf/ exotesta	2-DE, MALDI TOF-TOF	Kaempferol, kaempferol-3-O-glucoside, luteolin, quercetin, ginkgolide C, isoquercitrin, quercetin 3-galactoside, quercitrin, catechin	(Zheng et al., 2015)
	Uv-B Radiation	*Caththus roseus*		Leaf	Comparative gel-free proteomics	Hydroxy geraniol oxidoreductase, alkaloid biosynthesis	(Zhu et al., 2015)
	Uv-B Radiation	*Lonicaranera japonica* Thunb		Buds/ Flowers	2-DE, MALDI-TOF/TOF-MS	Sedoheptulose-1,7-bisphosphatase, 2,3-bisphosphoglycerate-independ ent phosphoglycerate mutase, transaldolase, alanine aminotransferase, 1-deoxy-D-xylulose 5-phosphate reductoisomerase, heat shock protein 70-2, Helicase, C-terminal, ubiquitin-like protein oxygen-evolving enhancer protein 1-2, urease accessory protein G, 20S proteasome alpha subunit E2 isoform 2, peroxisomal 2,4-dienoyl CoA reductase, Cytoplasmic aconitate hydratase, oxygen evolving enhancer protein 1-2-chloroplastic	(Zhu et al., 2017)
	Uv-B Radiation	*M. himalaica*,		Leaf	iTRAQ	The chalcone synthase enzymes, hormone signaling systems and phosphatidylinositol including auxin, abscisic acid, jasmonic acid	(Gu et al., 2018)
	Uv-B Radiation	*Lonicera japonica*		Leaf	2DE, MALDI-TOF/TOF MS	Enzymes involved in metabolite biosynthesis and defense mechanisms, Enhanced levels of caffeoylquinic acids and iridoids, increase in DXR (one-deoxy-D-xylulose 5-phosphate reductoisomerase) and EPSPS (five-enol-pyruvyl shikimate-phosphate synthase) production	(Zhang et al., 2013)
	Uv-B Radiation	*Amaranthus hypochondriacus*		Leaf	LC/ESI‐MS/MS	Up-regulation of chloroplast chaperonins, down-regulation of cytochrome b, Rubisco large subunit, the ascorbate peroxidase and oxygen-evolving complexes	(Huerta-Ocampo et al., 2009)
	Dark Treatment	*Clematis terniflora*,		Leaf	SDS–PAGE, LC–MS	Mitochondrial membrane permeability, the mitochondrial electron transport chain (mETC), photorespiration, and the tricarboxylic acid cycle	(Tao et al., 2022)
Drought	Water Deficit	*Scutellaria baicalensis* Georgi		Root	2-DE, MALDI-TOF MS	Electron transporter, putative, Adenosylhomocysteinase, Chloroplast heat shock protein 70B, d-3-phosphoglycerate dehydrogenase, Alpha-tubulin	(Yuan et al., 2012)
	Water Deficit	*chrysanthemum*		Leaf		Receptor-like cytosolic kinases CLE25, NCED3, CmWRKY10,	(Jaffar et al., 2016)
	Osmotic Stress	*Lepidium draba*		Sprouts	2−DE, MALDI-TOF/TOFMS	Ribulose bisphosphate carboxylase/oxygenase activase A, partia, Oxygen-evolving enhancer protein 1–2, chloroplastic, Aminomethyltransferase, mitochondrial, Endopeptidase La Ribulose-1,5-bisphosphate carboxylase/oxygenase large subunit, partial (chloroplast), Peptidyl-prolyl cis–trans isomerase CYP38, chloroplastic	(Jamshidi Goharrizi et al., 2020)
	Drought Stress	*Dendranthema grandiflorum*		Leaf	2-DE, MALDI-TOF/TOF MS	Glu S.griseus protease inhibitor-like, vacuolar protein sorting-associated protein 53 A isoform, zinc finger AN1 and C2H2 domain-containing stress-associated protein 16-like, RNA polymerase B, and probable disease resistance protein	(Sahithi et al., 2021)
	Water Deficit	*Taxillus chinensis*		Seeds	TMT-labeling, LC-MS/MS	Calvin cycle-associated and glycolysis-related proteins, fructose aldolases and glucose pyrophosphorylase,	(Pan et al., 2022)
Temperature	Heat Stress	*Pinellia ternata*		Leaf	2-DE, MALDI-TOF/TOF MS	HSP21, heat shock protein 17.9, small heat shock protein	(Zhu et al., 2013)
	Heat Stress	*Momordica charantia* L. var. Hong Kong Green		Fruit	2D-DIGE, nano-HPLC-MS/MS	UDP-glucose pyrophosphorylase (UGPase), Phosphoglycerate kinase (PGK), Quinoneoxidoreductase, Heat shock protein, 18.1 kDa class I (sHSP), Heat shock protein, 18.2 kDa class I (sHSP)	(Ng et al., 2014)
	Heat Stress	*Panax ginseng Meyer*		Leaf	Label-free quantitative proteome	Receptor chaperones, G-proteins, calcium-signaling proteins, transcription factors, structural-binding, and transfer/carrier proteins	(Kim et al., 2019)
	Heat Stress	*Maca (Lepidium meyenii Walp)*		Leaf	TMT	HSPs proteins	(Wang et al., 2020)
	High Temperature	*Dendranthema grandiflorum*		Leaf	LC-MS/MS	Eukaryotic translation initiation factor 3, eukaryotic translation initiation factor 4B3-like, chloroplast translation initiation factor IF-2, fructose kinase	(Li et al., 2021)
	Cold Stress	*R. chrysanthum*		Leaf	Proteomics (iTRAQ) techniques	Expressed two heat shock proteins (Hsp18 and Hsp70	(Zhang et al., 2023)
	Cold Stress	*Calendula officinalis*		Leaf	Two dimensional (2D) and MALDI-TOF-MS	Proteins corresponding to photosynthesis, respiration, stress resistance, antioxidant defense, plant development and signal transduction	(Jan et al., 2023)
	Chilling Stress	*T. chinensis*		Seeds		Involved at biosynthesis, transport, energy, and cellular metabolism	(Pan et al., 2025)
Heavy Metals	High Copper	*Cannabis sativa*		Roots		Up-regulated putative auxin-induced proteins such as plant growth regulator-related proteins, formate dehydrogenase (FDH), the scavenging system proteins represented by aldo/keto reductase, enolase and elicitor inducible; stress resistance proteins cyclophilin and putative peroxidase; and suitable growth regulator proteins glycine-rich RNA binding protein (GRP)	(Bona et al., 2007)
	Cadmium Stress	*Agrostis capillaris L*		Metal stress	2-DE and identification by LC-MS/MS	Cytochrome b6-f complex, oxygen evolving enhancer protein 1 (OEE), chlorophyll a-b binding proteins, and RuBisCO, redox enzymes, isocitrate dehydrogenase, cysteine/methionine synthases and chaperones	(Hego et al., 2016)
	Cadmium Stress	*Amaranthus hybridus* L.		Root	2-DE, MALDI-TOF/TOF-MS	ATP synthase subunit beta, mitochondria, Enolase, Eukaryotic elongation factor 1A, S-adenosyl-L-homocysteine hydrolase, Plastid-lipid-associated protein, chloroplast precursor, putative, Phytochrome C, partial UDP-glucose 6-dehydrogenase, Lignin-forming anionic peroxidase precursor, putative, Actin, Glutathione S-transferase, Dicer-1, putative	(Jin et al., 2016)
	Cadmium Toxicity	*Crocus sativus* L		Leaf	2-DE, MALDI-TOF-TOF-MS	5methyltetrahydropteroyltriglutamate-homocysteine methyltransferase, Glutamine synthetase, S-adenosylmethionine synthetase 1, Glyceraldehyde-3-phosphate dehydrogenase Chaperonin, Stromal 70 kDa heat shock-related protein, chloroplastic, Ferredoxin-NADP reductase, leaf isozyme	(Rao et al., 2017)
	Cu Stress	*Eucalyptus camaldulensis*		Seedlings	2-DE, MS	Stress proteins, metabolism and regulatory proteins	(Alotaibi et al., 2019)
	High Copper	*Hyoscyamus albus* L		Root	2-DE, MALDI-QIT-TOF	Pyrophosphate-fructose 6-phosphate 1phosphotransferasebeta-subunit, Enolase, S-adenosylmethionine synthase, Ferredoxin-nitrite reductase, Heat shock cognate 70 kDa protein	(Sako et al., 2016)
	High Copper	*Eucalyptus camaldulensis*		Leaf	2-DE, MALDI-TOF-TOF-MS	Elongation factor tu, c-repeat binding factor, Glyceraldehyde-3-phosphate dehydrogenase, fructose-bisphosphate aldolase, sucrose synthase Ribulose bisphosphate carboxylase large chain,	(Alotaibi et al., 2019)

#### 2.5.2 Light spectrum and low/high light intensity

Light is a critical environmental factor influencing plant growth and development. The balance between photoreception and intensity directly impacts photosynthetic efficiency. Under natural conditions, plants often receive suboptimal light, resulting in photosynthetic imbalance. Both excessive and insufficient light can damage protein complexes involved in electron transport, disrupting NADPH and ATP availability. To adapt to these stresses, photosynthetic organisms employ various photoprotective mechanisms and protein modifications. Thylakoid membrane protein complexes, such as photosystems (PS) I and II, their light-harvesting antennae (LHC I and II), cytochrome (Cyt) b6f, and the ATP-synthase complex, work in coordination to absorb light energy and convert it into a chemical form essential for plant survival (Wietrzynski et al., 2020). Protection mechanisms have evolved in PSII to counteract ROS generation by tuning redox processes and inhibiting damage to the photosystem (Brinkert et al., 2016). Ultraviolet (UV) radiation is the most impressive ray of light that impacts every level of plants’ biological organization and alters the biochemistry of plants. Ultraviolet radiation activates the photoregulatory pathway (Brown and Jenkins, 2008), damages photosynthetic pigments (Frohnmeyer and Staiger, 2003), enhances ROS production (Trentin et al., 2015), and elicits secondary metabolite production in medicinal plants (Binder et al., 2009). The impact of light or UV radiation on particular biological pathways and processes can be described by alterations in the contents of proteins that have a functional role in the performance of photosynthesis.

Plants activate defense strategies by regulating specific genes and accumulating UV-absorbing compounds in response to light/UV stress (Frohnmeyer and Staiger, 2003). Modern pharmacological studies have confirmed the high medicinal value of *Lonicera japonica*. The main molecular processes involved in its development when exposed to UV radiation were analyzed by comparative proteomics. Studies have shown that UV radiation significantly increases the levels of proteins involved in antioxidant activity, energy and carbohydrate metabolism, and secondary metabolite content in *Lonicera japonica* flower buds. These alterations in bioactive components, primarily secondary metabolites, influence both the quality and quantity of the resulting medicinal materials (Zhu et al., 2017). Similar results have also been reported in association with the effect of blue/red light on *Scrophularia kakudensis* proteomic profiles, referring to up-regulation of carbohydrate metabolism, photosynthesis, and stress responses as well as stimulation of secondary metabolites with medicinal value via activated stress alleviation mechanisms (Manivannan et al., 2021). Comparative proteomics using 2-DE combined with MALDI-TOF/TOF MS was conducted to identify key enzymes involved in metabolite biosynthesis and defense mechanisms in UV-irradiated *Lonicera japonica*. Findings suggested that UV stress activated a complex defense system, leading to differential protein accumulation across various molecular processes, including photosynthesis, secondary metabolite biosynthesis, transport, carbohydrate and energy metabolism, cell wall dynamics, and lipid metabolism. Enhanced levels of caffeoylquinic acids and iridoids were recognized as secondary metabolites with antioxidant and UV-absorbing properties. Additionally, an increase in DXR (one-deoxy-D-xylulose 5-phosphate reductoisomerase) and EPSPS (5-enolpyruvylshikimate-3-phosphate synthase) production, as two UV-responsive key enzymes, provides more precursors for secondary metabolite biosynthetic pathways following exposure to UV stress (Zhang et al., 2013).

High radiation may disrupt the balance between the energy absorbed through the photophysical processes of PSII and the energy utilized for carbon assimilation (Joshi et al., 2011). The major function of chloroplasts, namely photosynthesis, could be severely suppressed by enhanced UV-B irradiation. To prevent UV-B’s detrimental effects on photosynthesis, flavonoids are actively expressed, acting both as an internal filter against this harmful radiation and participating in the antioxidant defense system (Ferreyra et al., 2021). Zhu et al. (2015) also discussed the negative impact of UV-B irradiation and dark incubation on photosynthesis in *Catharanthus roseus* leaves by reporting the diminution in abundance of sedoheptulose-1, 7-bisphosphatase, chlorophyll a/b binding protein, serine glyoxylate aminotransferase 3, ribulose bisphosphate carboxylase/oxygenase, and light-harvesting complex II proteins. The declining production of RuBisCO and PSII-related proteins, in both whole leaves and chloroplasts, implies the adverse effect of UV-B irradiation on the photosynthetic system efficiency. Obtained data from a comparative proteomic approach confirmed that augmented UV-B radiation exposure up-regulated antioxidant, stress-responsive proteins, and flavonoid biosynthesis enzymes, while reducing photosynthesis rate in *Ginkgo biloba* traditional medicinal plant (Zheng et al., 2015). *Catharanthus roseus* synthesizes a diverse range of indole alkaloids, which exhibit notable pharmaceutical properties. Comparative gel-free proteomics revealed a high abundance of 10-hydroxy geraniol oxidoreductase, involved in the biosynthesis of indole alkaloids in *C. roseus* leaves. The UV-B irradiation and darkness induced alkaloid biosynthesis by altering related metabolic pathways and therewith significantly boosted indole alkaloids, including ajmalicine, vindoline, catharanthine, and strictosidine production (Zhu et al., 2015). UV-B radiation could have a remarkable positive effect on the medicinally active substances, namely rotenoids, flavonoids, and coumarins, as the secondary metabolite. To focus on the role of UV-B radiation in regulating the metabolism of *M. himalaica*, a Tibetan medicinal plant, an iTRAQ proteomics approach was used. It was found that under exposure to UV-B radiation, up-regulation of DAPs such as the chalcone synthase enzymes improved the biosynthesis of rotenoid through the hormone signaling systems and phosphatidylinositol, including auxin, abscisic acid, jasmonic acid, and calcium signals (Gu et al., 2018). Mitochondrial proteomics was employed to investigate the response mechanism of *Clematis terniflora*, a medicinal plant from the Ranunculaceae family, to UV-B radiation and dark treatment. DAPs were primarily linked to mitochondrial membrane permeability, the mitochondrial electron transport chain (mETC), photorespiration, and the tricarboxylic acid cycle, to minimize energy consumption and maintain energy balance under stress. Additionally, alternative oxidases played a role in regulating intracellular oxygen balance by engaging other oxygen-consuming pathways (Tao et al., 2022).

#### 2.5.3 Drought

Water deficit is one of the most intense environmental restrictions on plant productivity. Drought stress disturbs plant water relations and diminishes water-use efficiency, root proliferation, and eventually yield (Kazemi Oskuei et al., 2023). Drought conditions also affect plants’ metabolic processes and lead to cellular damage (Perlikowski et al., 2022; Sahithi et al., 2021). However, concerning spice and medicinal plants, the situation is different. Drought has a significant effect on the growth and secondary metabolic pathways of medicinal plants. Exposure to drought stress reduces medicinal plants’ biomass production as well, yet enhances the contents and quality of important compounds (Shil and Dewanjee, 2022). Various response strategies in plants have been proposed at physiological and biochemical levels to cope with drought stress (Farooq et al., 2009).

To trigger the defense mechanisms immediately during any stress, various stress-responsive proteins remain active in plants. For the relative protein profiling of chrysanthemum (*Dendranthema grandiflorum*) Sahithi et al. (2021) performed 2-DEs combined with MALDI-TOF MS. Most of the DAPs under drought stress conditions were mostly related to flower development and stress response/defense. An increase in stress response proteins’ content was associated with reduced reactive oxygen species (ROS) and redox reaction regulation to protect the cell from oxidative damage and maintain homeostasis (Sahithi et al., 2021).

The seeds of *Taxillus chinensis*, belonging to *Taxillus*, a genus of parasitic plants that are important herbs in the Chinese pharmaceutical industry, are sensitive to dehydration. Dehydration stress induces oxidative damage in seeds. Proteomics analysis using TMT-labeling and LC-MS/MS identified DAPs primarily localized in the chloroplast, playing key roles in photosynthesis, signal transduction, and energy metabolism. Increased levels of Calvin cycle-associated and glycolysis-related proteins, such as fructose aldolases and glucose pyrophosphorylase, contribute to energy production and enhanced dehydration tolerance in *Loranthus* seeds (Pan et al., 2022). The up-regulation of chloroplast chaperonins involved in refolding and protein complexes protection, along with the down-regulation of cytochrome b, Rubisco large subunit, the ascorbate peroxidase and oxygen-evolving complexes, as mitochondrial proteins, disclosed the central role of chloroplasts and mitochondria in abiotic stress adaptation in *Amaranthus hypochondriacus* L (Huerta-Ocampo et al., 2009).

Up-regulation of GA-responsive protein and anthranilate synthase as indoleacetic acids (IAA)-related proteins in leaves of *Scutellaria baicalensis*, a traditional Chinese herbal plant exposed to water deficit, was detected by proteomic analysis, demonstrating the effect of water deficits on flavonoid accumulation through hormonal metabolism regulation. The great accumulation of flavonoids as active compounds affects the high quality of this herbal medicine (Yuan et al., 2012). In addition, an appropriate degree of drought promotes baicalin accumulation, stimulating the production of key biosynthetic enzymes (L. Cheng et al., 2018). The proteomic profiles of drought-stressed Mulberry Trees (*Morus alba* L.) showed DAPs mostly enriched in the sucrose-related metabolic pathway (Liu et al., 2019a).

The differential regulation of proteins involved in cell wall strengthening, signal transduction, gene regulation, and cellular detoxification affects the molecular mechanism behind drought stress tolerance (Kottapalli et al., 2009). Proteomic analysis of *Lepidium draba* under drought stress revealed differential regulation of key proteins involved in energy metabolism and photosynthesis, including rubisco activase A, RuBisCO large subunit, aminomethyl transferase mitochondrial-like proteins, endopeptidase La protein, and oxygen-evolving enhancer protein 1–2 (OEE1-2). Additionally, the two-component system (TCS), comprising histidine kinase proteins (HKs), histidine phosphotransfer proteins (Hpts), and response regulator proteins (RRs), played a role in signal transduction and environmental sensing. Glycosyltransferase contributed to plant development, while glutamate 5-kinase, a component of the proline biosynthesis pathway, likely enhanced drought tolerance in *L. draba* (Jamshidi Goharrizi et al., 2020). Comparative proteomics of chrysanthemum leaves under drought stress identified DAPs involved in flower development, stress signaling pathways, and secondary metabolic processes, including the regulation of the circadian rhythm, physiological transport, DNA synthesis, gene expression, and protein ubiquitination. These findings contributed to defining the key signaling networks, homeostatic regulation, and tolerance mechanisms, essential for *chrysanthemum*’s adaptation to drought stress (Sahithi et al., 2021). In the view of Jaffar et al. (2016), the overaccumulation of CmWRKY10, which is known as DNA DNA-binding protein in chrysanthemum, was induced by drought to behave as a positive regulator of stress-related genes with a role in ABA signaling and cellular ROS production, and improve its drought tolerance. Receptor-like cytosolic kinases CLE25 contribute to transmitting drought signals to downstream targets, leading to the activation of NCED3, a crucial enzyme in abscisic acid (ABA) biosynthesis (Takahashi et al., 2018). Therefore, the involvement of protein kinases in stress signal transduction is co-regulated with plant responses to drought stress in all aspects.

#### 2.5.4 Temperature

Temperature is one of the most critical abiotic stresses that profoundly affects plant growth and productivity (Parankusam et al., 2017). Both heat stress (high temperatures) and cold stress (low temperatures) significantly disrupt the physiological processes of plants, including protein synthesis, metabolism, and cellular integrity. Each medicinal plant species has its own optimal temperature range; extreme variations can quickly disrupt its cellular structures, macromolecules, and, in particular, valuable secondary metabolites (Alum, 2025). Hence, medicinal plants similar to crops have evolved different adaptation mechanisms to withstand adverse conditions for optimal growth (Niu and Xiang, 2018). The induction of variety cellular phenomena, namely membrane fluidity, metabolite, osmolyte concentrations, photosynthesis, carbon assimilation, and redox status, constitutes responses to heat stress (Niu and Xiang, 2018). According to the Sher et al. (2024) the alteration of the post-translational, transcriptional, and translational mechanisms, followed by changes in signaling pathways, is a key strategy for combating heat stress in medicinal plants. High or low temperatures disrupt protein stability, specific enzyme functions, and perturb metabolism. Therefore, the identification of protein profiles of medicinal plants by proteomics in response to extreme temperatures is crucial to understand the molecular mechanisms that underpin response strategies in medicinal plants’.

The heat shock transcription factors (HsfA1s), which are the master regulators of the heat stress response (HSR), acquire thermotolerance in plants through interaction with heat shock proteins (HSPs). So, the enhancement in the level of HsfA1s and HSPs (HSP70 and HSP90) leads to adaptation and heat stress tolerance in medicinal plants (Ohama et al., 2016). Gouda et al. (2024) reported that the optimal response in which cells survive the heat stress was the elevation of HSP levels as heat-responsive proteins. In addition, the upregulation of HSPs prevented other cellular proteins from damage caused by heat stress.

The evaluation of heat-responsive proteins in *P. ternata* through 2-DE followed by MALDI-TOF/TOF MS showed that in the protein expression pattern, more than 20 proteins were up- and down-regulated in response to heat stress. Several tolerance-related proteins with various functions were identified, including small HSPs, RNA processing proteins, photosynthesis proteins, protein degradation proteins, and defense proteins (Zhu et al., 2013). It was reported that heat stress reduced the photosynthetic efficiency of *Ginseng* (*Panax ginseng* Meyer) 48 h after treatment, and modulated 847 differentially abundant proteins, which were identified by label-free quantitative proteome analysis in response to stress. These proteins with increased abundance were mainly associated with antioxidant and translation-regulating activities, whereas the proteins related to the receptor chaperones, G-proteins, calcium-signaling proteins, transcription factors, structural-binding, and transfer/carrier proteins activities exhibited reduced abundance (Kim et al., 2019). DAPs in the leaves of *Dendranthema grandiflorum* ‘Jinba’ under high temperature stress were investigated using label-free quantitative proteomics techniques and LC-MS/M. It was demonstrated that most of the DAPs were involved in energy metabolism pathways, protein metabolism, and heat shock, as well as some of them had a correlation with heat resistance in chrysanthemum (Li et al., 2021b). Proteomic analysis of Maca (*Lepidium meyenii* Walp) showed that the levels of 300 proteins, in particular, HSP proteins, which regulate protein quality, were differentially changed in response to the high temperature stress. HSPs were significantly up-regulated to protect other proteins from being denatured. Moreover, a variety of transcription factors, including MBF1C, HSFA2, AF1, WRKY70, and HY5, that take part in the inspection of HSR-related genes, ROS scavengers, and enzymes, were regulated by these HSPs (Wang et al., 2020).

Likewise, proteomic analyses revealed that cold acclimation enhanced the cold tolerance of medicinal plants by promoting the biosynthesis of proteins that participated in ROS scavenging, photosynthesis, energy metabolism, carbohydrate metabolism, protein metabolism, and cofactor biosynthesis (Dugganaboyana et al., 2023). By using MALDI-TOF/TOF-MS, Shen et al. (2021) found some proteins in G8, such as TIM, ATPB, and LEA in both cold-acclimated and non-acclimated *S. apetala* seedlings, which act as common responsive proteins in response to chilling stress. Also, they suggested that CAB, eEF-G and APX1 proteins function as the hub proteins in regulating the stress response. Zhang et al. (2023), using integrated transcriptomics (RNA-seq) and proteomics (iTRAQ) techniques, have revealed the enrichment of some pathways comprising antioxidant activity and carbohydrate metabolism through the induction of six different genes (GLUST, GO1, RPE3, P5PI3, RbcS, and POD4) and differentially expressed two heat shock proteins (Hsp18 and Hsp70) in two CfT lines of (cold-resistant) *R. chrysanthum* exposed to cold stress. Comparative proteomics of *Ammopiptanthus mongolicus* leaves under cold stress identified differentially accumulated proteins involved in photosynthesis in chloroplasts, reactive oxygen species scavenging, defense system, protein synthesis, protein folding, and protein degradation (Zheng et al., 2023). The findings of Pan et al. (2025) revealed that the differentially expressed proteins were predominantly involved in biosynthesis, transport, energy, and cellular metabolism in *T. chinensis* seeds under chilling stress. There was an investigation of the cold stress tolerance mechanism in *Calendula officinalis*, assessing 2-DE and MALDI-TOF-MS proteomic analyses in exposure to 4 °C at different time intervals. It was indicated that *Calendula officinalis*, which is an important medicinal plant, can withstand cold stress due to the involvement of proteins corresponding to photosynthesis, respiration, stress resistance, antioxidant defense, plant development, and signal transduction. Furthermore, the main pathway for protecting this plant against cold stress were introduced as the Glutathione-ascorbate pathway and different antioxidants (Jan et al., 2023).

#### 2.5.5 Heavy metals

One of the major abiotic stresses that leads to serious effects in plants is heavy metal (HM) toxicity. Heavy metal (HM) term is generally used for an extensive range of metal(loid)s that are natural constituents of soils including nickel (Ni), cobalt (Co), cadmium (Cd), copper (Cu), chromium (Cr), lead (Pb), zinc (Zn), boron(B), and arsenic (As); the presence of these toxic ions seriously alters the physiological and metabolic processes of living organisms. However, most of the heavy metal(loid)s are indeed micronutrients and/or elements essential in small quantities for the functional behaviors of many proteins associated with supporting the normal growth and development of plants (Alloway, 2013). Generally, an extensive consequence of HM ions’ entrance into cells is interacting with vital constituents, inactivating the enzymes, interacting with sulfhydryl groups of proteins, displacement of essential cations from their specific binding sites, and excessive ROS generation contribute to oxidative damage in lipids, nucleic acids, and proteins, and ultimately inhibiting plant growth and causing cell death (Hossain et al., 2012).

To withstand excess HMs, plants use diverse strategies as coordinated homeostatic mechanisms leading to uptake limitation or sequestration, as well as constitutive and adaptive mechanisms.

The compatible solutes, metallothionein, phytochelatins, and secondary metabolites are part of various molecules that participate in attenuating the negative impact of HMs. Hence, gaining information at the translational and/or post-translational levels, and identifying the function of genes/proteins, are fundamental steps in understanding the molecular mechanisms of heavy-metal stress responses and developing tolerant transgenic plants that are capable of detoxification or toxic elements removal from soils (Riyazuddin et al., 2021). Comparative or quantitative proteomics studies represent an efficient platform for the recognition of biologically functional complex protein networks in organisms subjected to constraints. Proteomics provides a preferable concept of the specific mechanisms and biochemical pathways related to tolerance mechanisms to metal stress and cellular detoxification (Hossain and Komatsu, 2013).

Certainly, limited research has been performed on the effects of heavy-metal stress on pharmacologically active substances and the protein abundance of medical plants in the last decades (**Table 3**). *Cannabis sativa*, which is an important, tolerant herbaceous species from central Asia, has a high capability to cope with high metal concentrations in soil. The proteomic analysis of *Cannabis sativa* roots exposed to high Copper concentration (Bona et al., 2007) exhibited that the Copper treatment up-regulated putative auxin-induced proteins such as plant growth regulator-related proteins, formate dehydrogenase (FDH), which plays a role in maintaining a reduced environment, and the scavenging system proteins represented by aldo/keto reductase. While stress-responsive proteins enolase and elicitor inducible stress resistance proteins cyclophilin and putative peroxidase, and suitable growth regulator proteins glycine-rich RNA binding protein (GRP) were down-regulated.

The high accumulation of HMs inhibited the plant’s growth and its photosynthetic product. The separation of proteins from *Agrostis capillaris* L. under metal stress with 2-DE and identification by LC-MS/MS demonstrated that high Copper concentration severely disrupted the photosynthesis apparatus and impacted both light-dependent and -independent photosynthetic pathway-related proteins like cytochrome b6-f complex, oxygen-evolving enhancer protein 1 (OEE), chlorophyll a-b binding proteins, and RuBisCO. The enhancement of redox enzymes, isocitrate dehydrogenase, cysteine/methionine synthases, and chaperones in *Agrostis capillaris* L. was related to Cu detoxification and tolerance of this metallicolous plant (Hego et al., 2016).

Alotaibi et al. (2019) characterized the molecular mechanism underlying the impact of Cu stress on *Eucalyptus camaldulensis* and the achievement of homeostasis in response to this metal. As attested by the proteomics results, altered proteins were involved in photosynthesis, protein metabolism, and regulation; special stress-related proteins further assisted *E. camaldulensis* seedlings in handling Cu exposure. Comparative proteomic analysis of *Hyoscyamus albus* L. seedlings exposed to copper revealed that proteins involved in proteasome were decreased in abundance whilst the energy supply and anabolism proteins were increased as well as newly generated proteins acting as Cu-binding reservoirs for deposition of additional Cu. It was indicated that high levels of Cu increased the activity of respiration and promoted the propagation of *H. albus* roots by enhancing proteins associated with protein synthesis, carbohydrate metabolism, and ATP synthesis (Sako et al., 2016).

According to proteomic analysis, arsenic bioaccumulation negatively impacted most of the proteins related to energy and carbon metabolism inducing a metabolic disorder in the *Acacia farnesiana* (Sweet Acacia) medicinal plant utilized in Mexican traditional medicine. Since *Acacia farnesiana* under Arsenic stress requires more energy to maintain cellular homeostasis, endosymbiosis with *Methylobacterium* sp. provided the required energy to cope with arsenic through up-regulation of carbonic anhydrase which promotes the photosynthetic activity. In addition, the up-regulated defense-related proteins were involved directly in the ASC-GSH cycle and ROS metabolism (Alcántara-Martínez et al., 2018). The substantial amounts of secondary metabolites in *Tetraena qatarensis* indicated its potential medicinal significance. MALDI-TOF/MS and in silico proteome analysis of lead-treated *Tetraena qatarensis* demonstrated DAPs having a role in ion and protein binding, transport, antioxidant activity, and abiotic stress response. In spite of the increased level of proteins linked with HSF1-mediated heat shock proteins regulation, glutathione metabolism, and cellular response as stress-regulating metabolic pathways, the role of the identified proteins, in particular, up-regulated glycine-rich protein (GRP) in Pb tolerance and or detoxification has not been clarified (Usman et al., 2022). Analysis of Cd-exposed soybean samples by 2-DE coupled with MS revealed DAPs associated with Cd-chelating pathways. Moreover, increased xylem lignification prevented translocation of Cd from the roots to the aerial parts by significantly up-regulation of lignin biosynthesis-associated proteins under Cd stress (Ahsan et al., 2012). Jin et al. (2016) submitted a report regarding higher Cd enrichment in *Amaranthus hybridus* L. roots in comparison with other organs as well as the identified DAPs by MALDI-TOF MS indicated that the redirection of root cell metabolism was the main survival mechanism of *A. hybridus* under Cd stress. Moreover, it was proven that tolerance and enrichment strategies were activated in *A. hybridus* through up-regulation of proteins related to protein metabolism (proteasome subunit alpha type and ClpC protease), energy metabolism (fructokinase, ATP synthase subunit beta, 2-phospho-D-glyceratehydrolase, enolase, and fructose-biphosphatealdolase), stress and defense (salt tolerance protein I, GST, and salt tolerance protein II) and signal transduction (wall-associated receptor kinase-like14 and phytochrome C) to tolerate Cd stress.

### 2.6 AI-assisted proteomics

Proteins are the main functional components of the cell; they control gene expression, support cell structure, and provide enzymatic machinery. Additionally, since most biomarkers and beneficial targets are proteins, proteomics focuses on four key aspects, namely sequence, structure, function, and expression, to offer a comprehensive understanding of the intricate interactions within plant cells (Aizat and Hassan, 2018). Despite the remarkable capabilities of proteomics, the vast complexity of the proteome poses a significant analytical challenge. Moreover, other “omics” technologies provide valuable insights into the molecular mechanisms governing plant responses, capturing various layers of gene expression and small molecule generation. As a result, these approaches are producing datasets that rival proteomics in size and often surpass them in multidimensional complexity (Mund et al., 2022). Formerly, results could be summed up in a few spreadsheets, but nowadays even individual projects with each omics generate data that require processing and interpreting in the best way. Traditional statistical models struggle to effectively process the immense complexity of multi-dimensional omics datasets, necessitating the development of advanced computational approaches for deeper insights into biological systems (Murmu et al., 2024). Key difficulties include real-time MS analysis, high-dimensional data interpretation, post-translational modification (PTM) site prediction and predicting protein-protein interactions from primary sequences. Additionally, automating hypothesis generation, understanding mutation effects on protein function and structure, repurposing existing drugs, identifying drug targets, and deeply integrating omics data require advanced computational strategies to navigate their complexity effectively (Stransky et al., 2023). To solve these challenges, recently Artificial Intelligence (AI) has appeared as a powerful tool with a new perspective. The superiority of AI in genomic prediction and integrative analysis of plant omics data, particularly by using decision tree-based ensemble models was highlighted recently (Gokalp and Tasci, 2019; Isewon et al., 2022). Deep learning models, graph neural networks, and machine learning algorithms were introduced as state-of-the-art AI techniques for predicting protein-drug binding affinity (Yang et al., 2024). The high accuracy was achieved by utilizing machine learning algorithms to simplify the identification of SRG genes in exposure to various stress conditions with the SVM model (Meher et al., 2022) and a novel AI approach entitled improved MPMO-based differential evolution (IMPMO-DE) was added to multi-objective protein structure prediction (Hong et al., 2024). The Percolator algorithm, one of the earliest and most widely adopted machine learning techniques in proteomics, enhances the identification of true peptide hits by analyzing multiple experimental sequence features. By leveraging statistical learning, it optimizes the number of accurate matches at a specified false discovery rate (FDR), improving the reliability of peptide-spectrum matching in MS-based proteomics (Käll et al., 2007). According to Granholm et al. (2014) Percolator by increasing the number of identified peptides in comparison with MS-GF highlighting machine learning algorithms power in peptide identification, cross-linked peptide search, intact protein search, and DIA-based peptide search. AI also supports the MS-based shotgun proteomics workflow. Reports indicate that the integration of MS-based proteomics with artificial intelligence, high-content imaging, and single-cell laser microdissection enhances molecular analysis, delivering reliable information that closely reflects functional dynamics (Mund et al., 2022). Arsenovic et al. (2019) reported that deep learning, a subset of AI, has demonstrated remarkable accuracy in analyzing plant proteome characteristics influenced by genotype-environment interactions. While the advantages of AI in extracting meaningful insights from extensive datasets are well recognized, the specific challenges of AI implementation in plant proteomics—particularly in medicinal plant studies—remain insufficiently explored. Addressing these limitations will be crucial in driving future advancements in the field.

## 3 Discussion

In conclusion, studying proteomics in medicinal plants has proven to be a significant avenue for understanding the complex biological mechanisms underlying their therapeutic properties. This review has highlighted the recent advancements and methodologies applied in medicinal plant proteomics, including such techniques as 2-DE, iTRAQ, and LC-MS/MS, which have enabled the identification of proteins and metabolic pathways vital for the bioactive compounds.

The findings from various proteomic studies have underscored the potential of proteomics to provide deeper insights into the functional proteins and metabolic networks that contribute to the medicinal properties of plants. These insights can pave the way for developing new drugs and improving medicinal plant strains to enhance their tolerance to environmental stresses.

Despite these advancements, challenges and limitations exist such as the need for more comprehensive genomic and protein interactions data. Future research should focus on integrating proteomics with other omics technologies to build a more holistic understanding of medicinal plant biology. Additionally, exploring the proteomic responses of medicinal plants to different environmental conditions and stresses could yield valuable information for sustainable cultivation practices. Combining proteomics with AI may enable a profound understanding of the molecular foundations of medicinal plants’ response systems and lead to more effective protection strategies.

In summary, proteomics offers significant potential for enhancing our understanding of medicinal plants and their medical applications. Continued research in this field is crucial for fully harnessing the therapeutic benefits of these natural resources.
